# Amplicon Sequencing of the *slpH* Locus Permits Culture-Independent Strain Typing of *Lactobacillus helveticus* in Dairy Products

**DOI:** 10.3389/fmicb.2017.01380

**Published:** 2017-07-20

**Authors:** Aline Moser, Daniel Wüthrich, Rémy Bruggmann, Elisabeth Eugster-Meier, Leo Meile, Stefan Irmler

**Affiliations:** ^1^Agroscope Bern, Switzerland; ^2^Laboratory of Food Biotechnology, Institute of Food, Nutrition and Health, ETH Zurich Zurich, Switzerland; ^3^Interfaculty Bioinformatics Unit, University of Bern and Swiss Institute of Bioinformatics Bern, Switzerland; ^4^School of Agricultural, Forest and Food Sciences HAFL, Bern University of Applied Sciences Zollikofen, Switzerland

**Keywords:** strain typing, semiconductor sequencing, *Lactobacillus helveticus*, natural whey culture, population composition

## Abstract

The advent of massive parallel sequencing technologies has opened up possibilities for the study of the bacterial diversity of ecosystems without the need for enrichment or single strain isolation. By exploiting 78 genome data-sets from *Lactobacillus helveticus* strains, we found that the *slpH* locus that encodes a putative surface layer protein displays sufficient genetic heterogeneity to be a suitable target for strain typing. Based on high-throughput *slpH* gene sequencing and the detection of single-base DNA sequence variations, we established a culture-independent method to assess the biodiversity of the *L. helveticus* strains present in fermented dairy food. When we applied the method to study the *L. helveticus* strain composition in 15 natural whey cultures (NWCs) that were collected at different Gruyère, a protected designation of origin (PDO) production facilities, we detected a total of 10 sequence types (STs). In addition, we monitored the development of a three-strain mix in raclette cheese for 17 weeks.

## Introduction

*Lactobacillus helveticus* belongs to the group of lactic acid bacteria (LAB) that are characterized by their ability to produce lactic acid through metabolizing lactose and other carbohydrates (Hammes and Hertel, 2006). A previous study has shown that *L. helveticus* is one of the predominant species in the natural whey cultures (NWCs) that are used for the production of Gruyère, a protected designation of origin (PDO) cheese (Moser et al., 2017). Gruyère PDO, produced in specific regions of Switzerland, is a smear-ripened, hard-textured cheese made from raw cow's milk (Eugster-Meier et al., 2017). It is likely that *L. helveticus* plays an important role in this cheese's ripening process, as has been shown for other cheese varieties produced using NWCs (Gatti et al., 2014). Desirable effects in cheese production, such as faster ripening and enhanced flavor development, have been associated with specific strains of *L. helveticus* (Drake et al., 1997; Jensen M. P. et al., 2009; Jensen and Ardö, 2010). In addition, biotechnologically important characteristics can differ considerably between different strains of *L. helveticus* (Fortina et al., 1998). Due to the biotechnological importance of this species, it is an advantage to differentiate and characterize the various strains, as this allows for a better understanding of the functional and ecological significance of this species.

Several molecular typing methods, such as randomly amplified polymorphic DNA (RAPD) PCR, repetitive sequence PCR (rep-PCR), pulsed field gel electrophoresis (PFGE), ribotyping, and multilocus sequence typing (MLST) have been used in the past to examine the diversity of *L. helveticus* isolates (Giraffa et al., 1998; Jenkins et al., 2002; Jensen M. et al., 2009; Sun et al., 2015). All these techniques require cultivation. This can lead to a skewed microbial profile, as the culture medium and incubation could favor strains that are well adapted to these cultivation conditions. We aimed to deploy a culture-independent approach in the analysis to learn more about the population diversity and dynamics of *L. helveticus* strains in a dairy ecosystem; to our knowledge, such a method has not yet been developed for these strains.

In this study, we report on a culture-independent PCR-based method for the strain typing of *L. helveticus*. By exploiting the genomic sequence data of *L. helveticus* strains, a single copy gene of the core genome that encodes a surface layer protein was found to display high genetic heterogeneity and could be used for strain typing. We established an amplicon based high-throughput sequencing method to assess the composition of *L. helveticus* strains in NWCs and to monitor the development of *L. helveticus* strains during cheese ripening. The findings clearly show that the method is a useful tool to assess *L. helveticus* diversity in various habitats.

## Materials and methods

### DNA preparation

Genomic DNA (gDNA) was extracted from bacterial cultures, NWCs, and cheese samples as described by Moser et al. (2017). DNA quantity was determined fluorometrically using the Qubit dsDNA BR Assay Kit (Thermo Fisher Scientific, Baar, Switzerland).

### Target detection

The genomes of 57 *L. helveticus* strains from the Agroscope culture collection and that of one *L. helveticus* strain from the Direct Vat Set culture LH-32 from Christian Hansen (Copenhagen, Denmark) were sequenced (Supplemental Table S1). The “TruSeq DNA PCR-Free LT Library Prep” (FC-121-3003, Insert size option: 350 bp) was used to prepare the DNA libraries. The libraries were indexed and pooled, before being sequenced in one lane on an Illumina HiSeq 3000 instrument to produce paired-end reads (151 × 151). Trimmomatic (version 0.33, options: SLIDINGWINDOW:4:8 MINLEN:127; Bolger et al., 2014) was used to trim the raw reads. The remaining reads were assembled using SPAdes (version 3.6.1, options: —careful—mismatch-correction—k 21, 33, 55, 77, 99, 127) and the assembly was scaffolded using SSPACE (version 3.0, default options; Boetzer et al., 2011). The scaffolds were selected for length and coverage as follows: scaffolds that were shorter than 200 bp, and those with a lower median coverage than 20% of the median read-depth of all scaffolds larger than 5,000 bp, were excluded. The resulting assemblies were annotated using Prokka (version 1.11; Seemann, 2014). To determine the orthologous gene clusters (OGCs) between the genomes, all predicted protein-coding sequences were translated and compared using BLASTP (version 2.2.29+, default parameters; Altschul et al., 1990) and clustered using Ortho-MCL (version 2.0.9, default parameters; Li et al., 2003). Single copy OGCs that were present in all genomes were ranked according to the number of unique sequences per OGC. Afterwards the corresponding nucleotide sequences were extracted and aligned using Clustal Omega (version 1.2.1). The alignments were then inspected and not aligned sequences were removed manually using the CLC Main Workbench software (Version 7.5.1, Qiagen, Switzerland). Finally, the remaining aligned sequences were checked by eye for nucleotide polymorphisms. Thereby, a CDS encoding a surface layer protein, named *slpH* in this report, was found to carry a high amount of polymorphisms and could be used as a target for DNA-based strain typing.

For the assignment of sequence types (STs), additional orthologous genes were retrieved from the NCBI GenBank database. Based on the nucleotide polymorphism present in these genes, the nucleotide sequences were assigned to STs (Table 1). *SlpH* genes with newly identified polymorphisms were deposited in the NCBI database and their accession numbers are listed in Table 1. The sequences of all of the different *slpH* genes are presented in the nucleotide sequence alignment in Figure S1. The nucleotide sequences of the ORFs from all STs were translated using the CLC Main Workbench version 7.5.1 (CLC bio, Aarhus, Denmark) and the resulting amino acid sequences were analyzed using InterProScan (Jones et al., 2014). Furthermore, the genomic context of the *slpH* gene was analyzed in 10 complete *L. helveticus* genomes from the NCBI database (CP002081, CP000517, CP003799, CP002429, CP002427, CP009907, CP011386, CP012381, CP016827, and CP020029). Therefore, the genomes were reannotated using Prokka (version 1.11; Seemann, 2014) and the orthologous genes were determined using Ortho-MCL (Li et al., 2003). The *slpH* genes and the surrounding genes of all of the 10 strains were visualized using the package ggplot2 in R (Wickham, 2009).

**Table 1:** *slpH* genes used in this study and their assignment to sequence types (STs).

***L. helveticus* strain**	**GenBank accession number:region**	**Assigned ST**	***slpH* group**
CNRZ32^#^	NC_021744:188864..190276	ST18	3
DPC4571^#^	NC_010080:185923..187353	ST16	1
R0052^#^	NC_018528:173786..175240	ST7	2
H9^#^	NZ_CP002427:158282..159730	ST5	2
H10^#^	NC_017467:184227..185654	ST24	3
KLDS1.8701^#^	NZ_CP009907:360112..361575	ST4	2
MB2-1^#^	NZ_CP011386:169834..171285	ST6	2
CIRM_BIA_951	HG530785:24314..25666	ST26	2
CAUH18^#^	NZ_CP012381:192832..194253	ST19	3
D75^#^	NZ_CP020029:1867932..1866469	ST33	2
M3	NZ_JRTS01000020:2926..4227	ST12	3
LMG_22464	NZ_JQCJ01000044:7029..8378	ST25	1
CIRM_BIA_953	NZ_CBUH010000081:863..2185	ST28	3
LH12	LSVI01000069.1:5275..6597	ST29	3
DSM20075	NZ_GG700752:369748..371067	ST30	3
FAM1450	MF401525	ST20	3
FAM22287	MF401526	ST21	3
FAM1213	MF401527	ST22	3
FAM2888	MF401528	ST23	3
FAM21790	MF401529	ST17	3
FAM22076	MF401530	ST1	2
FAM1182	MF401531	ST2	2
FAM22077	MF401532	ST3	2
FAM13019	MF401533	ST10	2
FAM17275	MF401534	ST9	2
FAM22156	MF401535	ST11	2
FAM20575	MF401536	ST8	2
FAM8102	MF401537	ST13	1
FAM21339	MF401538	ST14	1
FAM21456	MF401539	ST15	1
LH32	MF417547	ST27	3

# *Completely assembled genome*.

### Amplification of the *slpH* locus

The *slpH* sequence from *L. helveticus* CNRZ32 (Table 1) served as reference sequence for primer design. Based on the reference sequence, the primer pair LHslpF (5′-CAAGGAGGAAAGACCACATGA-3′) and LHslpR (5′-TGTACTTGCCAGTTGCCTTG-3′) that amplifies a 1,116-bp region was designed. Primers were designed using Primer3 (version 0.4.0; Untergasser et al., 2012). PCR was carried out on a Veriti® Thermal Cycler (Thermo Fisher Scientific, Baar, Switzerland) with the following conditions: 95°C for 2 min followed by 30 cycles of 95°C for 20 s, 60°C for 10 s, and 70°C for 30 s, and a final extension of 70°C for 7 min. If not otherwise specified, each PCR (25 μL) contained 50 ng of gDNA for cheese or NWC samples or 1 ng of gDNA for pure cultures, 5 pmol of each primer, 5 nmol of each dNTP, 31.25 nmol Mg_2_SO_4_, 0.02 U of KOD Hot Start Polymerase (Merck), and 1X buffer for KOD Hot Start Polymerase. PCR products were analyzed using the Agilent DNA 7500 kit on an Agilent 2100 bioanalyzer (Agilent Technologies, Waldbronn, Germany).

### Culture-dependent typing

An NWC sample (100 μL, sample *p* in Table 2) was plated on modified MRS plates containing 20 g L^−1^ of lactose instead of glucose (De Man et al., 1960). After incubation at 42°C for 48 h, 96 colonies were picked from the agar plates and suspended individually in 100 μL of TE buffer (pH 8.0) containing 10 mM Tris-HCl and 10 mM EDTA. A heat treatment (100°C, 8 min) was used to extract gDNA. After the heat treatment, the suspensions with the lysed cells were centrifuged at 5,000 g for 10 min. The supernatant containing the DNA was diluted tenfold in 10 mM Tris-HCl (pH 8.0) and examined by quantitative real-time PCR (qPCR) to identify colonies of *L. helveticus*. DNA from *L. helveticus* positive colonies was further examined by *slpH* PCR as described above. The PCR products were sent to Microsynth (Balgach, Switzerland) for amplicon purification and Sanger sequencing.

**Table 2:** Natural whey culture (NWC) and cheese samples used for *slpH* amplicon sequencing.

**Sample**	**Type**	**Origin**	**qPCR Cq value^1^**	**Log_10_ copies ml^−1^ (g^−1^)**	**n° of STs detected**	**PCR *Lgall***
a	NWC	Villarzel, Switzerland	16.56 (± 0.26)	7.08 (± 0.08)	4	Negative
b	NWC	Corcelles-le-Jorat, Switzerland	15.56 (± 0.19)	7.37 (± 0.06)	3	Negative
c	NWC	Brenles, Switzerland	17.57 (± 0.08)	6.79 (± 0.02)	3	Negative
e	NWC	Semsales, Switzerland	17.17 (± 0.15)	6.90 (± 0.05)	3	Negative
f	NWC	Le Crêt, Switzerland	15.25 (± 0.10)	7.46 (± 0.03)	3	Negative
g	NWC	Châtel-St. Denis, Switzerland	17.09 (± 0.11)	6.93 (± 0.05)	3	Negative
h	NWC	Autigny, Switzerland	17.16 (± 0.16)	6.90 (± 0.05)	3	Negative
i	NWC	Chénes, Switzerland	18.00 (± 0.21)	6.67 (± 0.06)	3	Negative
k	NWC	Les Monts de Travers, Switzerland	16.60 (± 0.06)	7.07 (± 0.01)	3	Negative
l	NWC	La Brévine, Switzerland	14.16 (± 0.20)	7.77 (± 0.06)	1	Negative
m	NWC	La Sagne, Switzerland	17.57 (± 0.08)	6.94 (± 0.03)	3	Negative
n	NWC	Le Cerneux-Péquignot, Switzerland	14.28 (± 0.25)	7.74 (± 0.07)	4	Negative
o	NWC	Orsonnens, Switzerland	13.68 (± 0.23)	7.91 (± 0.07)	5	Negative
p	NWC	La Praz, Switzerland	12.72 (± 0.06)	8.18 (± 0.01)	4	Negative
q	NWC	Penthéréaz, Switzerland	12.05 (± 0.12)	8.13 (± 0.03)	3	Negative
1	Cheese	Dairy pilot plant	14.66 (± 0.19)	8.51 (± 0.04)	2	Negative
2	Cheese	Dairy pilot plant	20.69 (±0.02)	6.94 (± 0.08)	3	Negative
3	Cheese	Dairy pilot plant	21.63 (±0.87)	6.5 (± 0.23)	3	Negative

1 *Cq, quantification cycles*.

*The population density of L. helveticus was quantified by qPCR. The PCR assay for L. gallinarum (Lgall) was negative for all samples examined. A negative PCR result means that L. gallinarum was not present at a population density of 2.45 × 10^2^ CFU mL^−1^ or higher*.

### PCR assays for *L. helveticus* and *L. gallinarum* species confirmation

Identification of *L. helveticus* isolates and quantitation of the population density of *L. helveticus* representatives in NWCs and cheese was performed by a qPCR method targeting the single copy *pheS* gene that encodes the alpha-subunit of the phenylalanyl-tRNA synthetase as described elsewhere (Moser et al., 2017). Briefly, a plasmid containing the target sequence is used for absolute quantification. The equation of the standard curve was used to estimate the copy number for cheese (gene equivalent per g) and NWCs (gene equivalent per mL). Similarly, the *pheS* gene of *Lactobacillus gallinarum* was used as a target to develop a PCR-based detection method for this species. Based on the *pheS* nucleotide sequence from *L. gallinarum* DSM 10532 (NCBI GenBank Accession Number AZEL01000044:72250…73299), the primers 5′-TCAGGACCTTGTACTACCTTGTAA-3′ and 5′-TGCTACTAAGGCTGAAATCGT-3′ were designed, which enable amplification of a 180-bp fragment. The assay was used to analyze NWC samples for the absence or presence of *L. gallinarum*. PCR assays were conducted in a final volume of 25 μL containing 300 nM of each primer, 200 μM of each dNTP, 1.5 mM Mg_2_SO_4_, 0.02 U of KOD Hot Start Polymerase (Merck), and 1X buffer for KOD Hot Start Polymerase (Merck). Each PCR contained 50 ng of genomic DNA for NWC samples and 1 ng of DNA for pure cultures. The amplicons were amplified under the following conditions: 95°C for 2 min, followed by 30 cycles of 95°C for 20 s, 59°C for 10 s, and 70°C for 4 s, and a final extension of 70°C for 5 min. The PCR products were examined with an Agilent DNA 1000 kit on an Agilent 2,100 Bioanalyzer (Agilent Technologies, Waldbronn, Germany). To evaluate the lower limit of detection, DNA was extracted from a ten-fold dilution series of a pure culture from *L. gallinarum* DSM 10532 that had been grown overnight in MRS broth at 37°C and for which the population density had been determined by plate-counting.

### *slpH* gene semiconductor sequencing

After *slpH*-specific PCR, amplicons were purified using the Qiaquick PCR Purification kit (Qiagen, Hombrechtikon, Switzerland). Purified amplicons were fragmented by sonication of 1 μg of DNA in 130 μL nuclease-free water at 50 W, 200 cycles/burst, and 20°C for 90 s on a Covaris M220 instrument (Covaris, Brighton, U.K.). The fragmented DNA was end-repaired and adapter-ligated using the Ion Xpress Plus Fragment Library Kit (Thermo Fisher Scientific) according to the manufacturer's instruction. Samples were barcoded using the Ion Xpress Barcode Adapter 1-16 kit (Thermo Fisher Scientific). After barcoding, the DNA was size-selected for 400 bp reads using an E-Gel® SizeSelect™ Agarose Gel (Thermo Fisher Scientific). The libraries were quantified by qPCR using the Ion Library Quantitation Kit (Thermo Fisher Scientific). Before preparing the template-positive ion sphere particles with the Ion PGM Hi-Q OT2 kit (Thermo Fisher Scientific), each library was diluted to 100 pM in 10 mM Tris-HCl (pH 8.0) and 0.1 mM EDTA, before being pooled. The sequencing was performed using either an Ion 314™ or 316™ chip and the Ion PGM Hi-Q Sequencing kit on an Ion Torrent sequencer (Thermo Fisher Scientific).

### Sequence data analysis

First, fastq files were generated via the Torrent Suite Software (Thermo Fisher Scientific) using the default parameters; then, the reads were quality trimmed with Trimmomatic (version 0.36, options: SLIDINGWINDOW:4:20 MINLEN:101; Bolger et al., 2014). Finally, reads containing the identifying subsequences for *slpH* group1 (GGCTACACT, GATCAATTAA, AGTGTAGCC, and TTAATTGATC), for *slpH* group2 (CCTTAATGTA, CTGACGATGT, TACATTAAGG, and ACATCGTCAG), and for *slpH* group 3 (ATTGGTTCAG, GGTGTTGCTA, CTGAACCAAT, and TAGCAACACC) were extracted, trimmed, grouped based on 100% sequence identity, and mapped against a reference database containing known STs. Sequences that were not identical to one of the reference sequences were assigned to a new ST. For this procedure, a Python script was developed. The script, its usage and the database for the *slpH* STs are available at: https://github.com/danielwuethrich87/helveticus_strain_typing.

### Validation

The gDNA from 10 different *L. helveticus* STs (Table 3) were pooled in equal amounts. The amplicons obtained using the *slpH*-specific PCR were subsequently sequenced on the Ion Torrent sequencer as described above. The experiment was repeated three times.

**Table 3:** The developed strain typing approach was tested with a mixture of 10 *L. helveticus* strains.

**Assigned ST**	**N° of reads**	**Relative abundance (%)**	**Corresponding strain**
ST13	826 (± 53)	13.93 (± 0.59)	FAM8105
ST15	732 (± 57)	12.40 (± 1.12)	FAM21456
ST10	462 (± 59)	7.75 (± 0.08)	FAM13019
ST11	423 (± 48)	7.11 (± 0.18)	FAM22155
ST8	365 (±31)	6.14 (± 0.19)	FAM20575
ST1	349 (± 42)	5.85 (± 0.12)	FAM22076
ST23	942 (± 193)	15.73 (± 1.00)	FAM23235
ST22	757 (± 112)	12.68 (± 0.28)	FAM1213
ST17	653 (± 137)	12.89 (± 0.74)	FAM22330
ST20	446 (± 46)	7.51 (± 0.30)	FAM1450

*Reads obtained after NGS were assigned to sequence types (STs) and the average relative abundance for each ST (± standard deviation, n = 3) was calculated. The corresponding strains were determined by comparing the read sequences to the genome sequences of the used strains*.

## Results

### Characteristics of the *slpH* locus

We manually inspected the single copy orthologs of 58 own-sequenced genomes of *L. helveticus*. One of the gene clusters encoding a putative surface layer protein, named *slpH* in this study, stood out from the others in terms of nucleotide sequence diversity and differentiated 15 STs. Other OGCs differentiated between 8 and 11 STs and were excluded from further analysis. The primer pair LHslpF/R was designed to amplify the *slpH* locus in *L. helveticus*. The specificity of the primer pair was tested using the gDNA of 18 *L. helveticus* strains and 20 other species of LAB (Table 4). By using the primer pair LHslpF/R, an amplicon with a size of approximately 1,252 bp (±60 bp) was obtained for all the *L. helveticus* and *L. gallinarum* tested. No amplicon was observed using gDNA from *Streptococcus thermophilus* and other LAB, including the more closely related *Lactobacillus kefiranofaciens kefiranofaciens* DSM 5016, *L. kefiranofaciens kefirgranum* DSM 10550, *Lactobacillus crispatus* DSM 10532, *Lactobacillus acidophilus* DSM 20079, and *Lactobacillus amylovorus* DSM 20531 (Table 4).

**Table 4:** Bacterial strains used for the PCR studies.

**Strain**	**Isolation source**	**PCR *slpH*^#^**	***slpH* group**	**ST**	**PCR *Lgall*^#^**
***Lactobacillus helveticus***					
FAM 1450^1^	Unknown	+	3	20	−
FAM 1476^1^	Unknown	+	2	11	−
FAM 22155^1^	Natural whey culture	+	2	11	−
FAM 21493^1^	Mixed strain starter	+	1	14	−
FAM 22081^1^	Unknown	+	3	20	−
FAM 21456^1^	Mixed strain starter	+	1	15	−
FAM 8104^1^	Raw milk cheese	+	1	13	−
FAM 8105^1^	Raw milk cheese	+	1	13	−
FAM 13019^1^	Natural whey culture	+	2	10	−
FAM 8627^1^	Unknown	+	2	1	−
FAM 21339^1^	Mixed strain starter	+	1	14	−
FAM 22076^1^	Natural whey culture	+	2	1	−
FAM 23235^1^	Unknown	+	3	23	−
FAM 20575^1^	Natural whey culture	+	2	8	−
FAM 22330^1^	Mixed strain starter	+	3	17	−
FAM 1213^1^	Unknown	+	3	22	−
FAM 1172^1^	Unknown	+	1	13	−
FAM 22079^1^	Natural whey culture	+	2	3	−
***Lactobacillus gallinarum***					
DSM 10532^T^^,^ ^1^	Chicken crop	+	3	−	+
FAM 1941^1^	Chicken crop	+	3	−	+
LMG 14751^1^	Chicken feces	+	−	−	+
LMG 14754^1^	Chicken feces	+	−	−	+
LMG 14755^1^	Chicken feces	+	−	−	+
LMG 18181^1^	Chicken intestine	+	−	−	+
LMG 22870^1^	Laying hen vagina	+	−	−	+
***Lactobacillus kefiranofaciens kefiranofaciens***					
DSM 5016^T^^,^ ^1^	Kefir grains	−	−	−	−
***Lactobacilus kefiranofaciens kefirgranum***					
DSM 10550^T^^,^ ^1^	Kefir grains	−	−	−	−
***Lactobacillus delbrueckii bulgaricus***					
DSM 20081^T^^,^ ^1^	Yoghourt	−	−	−	−
***Lactobacillus delbrueckii lactis***					
DSM 20072^T^^,^ ^1^	Emmental cheese	−	−	−	−
***Streptococcus thermophilus***					
DSM 20617^T^^,^ ^1^	Pasteurized milk	−	−	−	−
***Lactobacillus crispatus***					
DSM 20584^T^^,^ ^1^	Eye	−	−	−	−
***Lactobacillus casei***					
FAM 18121^1^	Gruyére PDO cheese	−	−	−	−
***Lactobacillus rhamnosus***					
CCUG 34291^1^	Human feces	−	−	−	−
***Lactobacillus fermentum***					
DSM 20052^T^^,^ ^1^	Fermented beets	−	−	−	−
***Lactobacillus paracasei paracasei***					
DSM 5622^T^^,^ ^1^	Milk products	−	−	−	−
***Lactobacillus acidophilus***					
DSM 20079^T^^,^ ^1^	Human	−	−	−	−
***Lactobacillus johnsonii***					
DSM 10533^T^^,^ ^1^	Human blood	−	−	−	−
***Lactobacillus amylovorus***					
DSM 20531^T^^,^ ^1^	Cattle waste corn fermentation	−	−	−	−

*PCR results are indicated with + if an amplicon was observed, and with − if no amplicon was observed, after PCR*.

T *Type strain*.

1 *Strains were from the Agroscope Culture Collection (Switzerland) (FAM); the German Collection of Microorganisms and Cell Cultures (DSM); the Belgian Coordinated Collections of Microorganisms (LMG); and the Culture Collection University of Göteborg (Sweden) (CCUG); DVS, Direct Vat Set culture from Chr. Hansen Holding A/S, Denmark*.

# *+ and − indicate presence (+) or absence (−) of an amplicon using the L. gallinarum PCR*.

The nucleotide sequences of the *L. helveticus* amplicons were determined by Sanger sequencing and subsequently compared to *slpH* sequences that had been extracted from 79 bacterial genomes consisting of 58 genomes of own-sequenced strains and 21 from the NCBI GenBank. We found that the sequences were identical to the one derived from the illumina read assembly (data not shown) and that 30 STs could be unambiguously discriminated (Supplemental Figure S1). Additionally, the amplicon obtained from the *L. gallinarum* DSM 10532 was determined. A BLAST search revealed that the amplified region showed 99% identity to the *lgsB* gene (GenBank accession number AY597262) of *L. gallinarum*, which encodes a surface layer protein.

**Figure 1:**
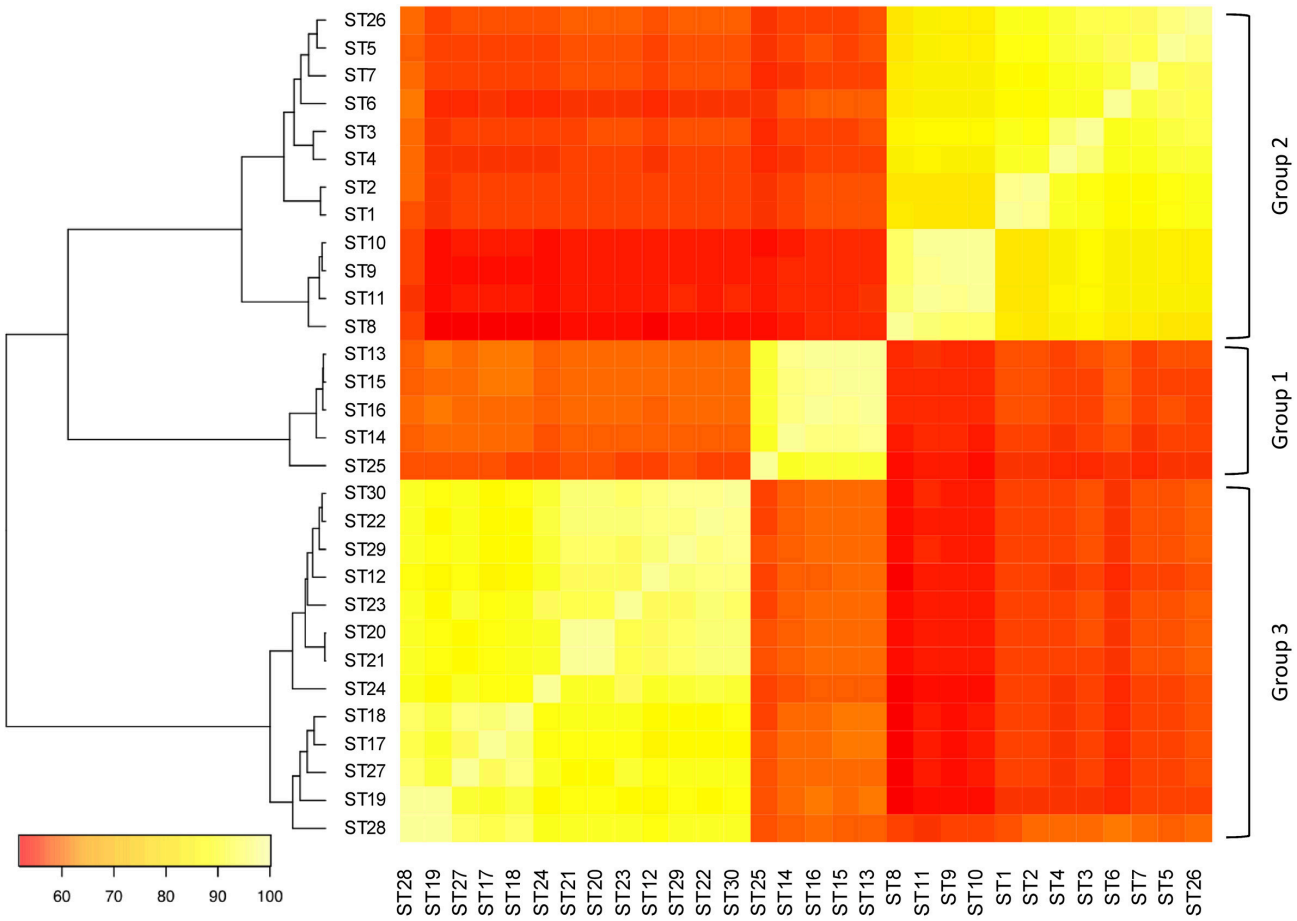
Pairwise comparison of the different sequence types (STs). The DNA sequences of the *in silico* derived amplicons from the different STs were compared to each other and the percent sequence identity was calculated for each pair. The color of a square between two STs indicates the percent sequence identity of the two STs. The color gradient ranges from white (100% sequence identity) to red (0% sequence identity).

A pairwise sequence comparison of 30 STs showed that the *slpH* sequences from *L. helveticus* clustered into three groups (Figure 1). Strains that clustered within a group shared, on average, 91.4 (±4.6)% sequence identity, whereas the identity between groups was, on average, 57.1 (±1.7)%.

**Figure 2:**
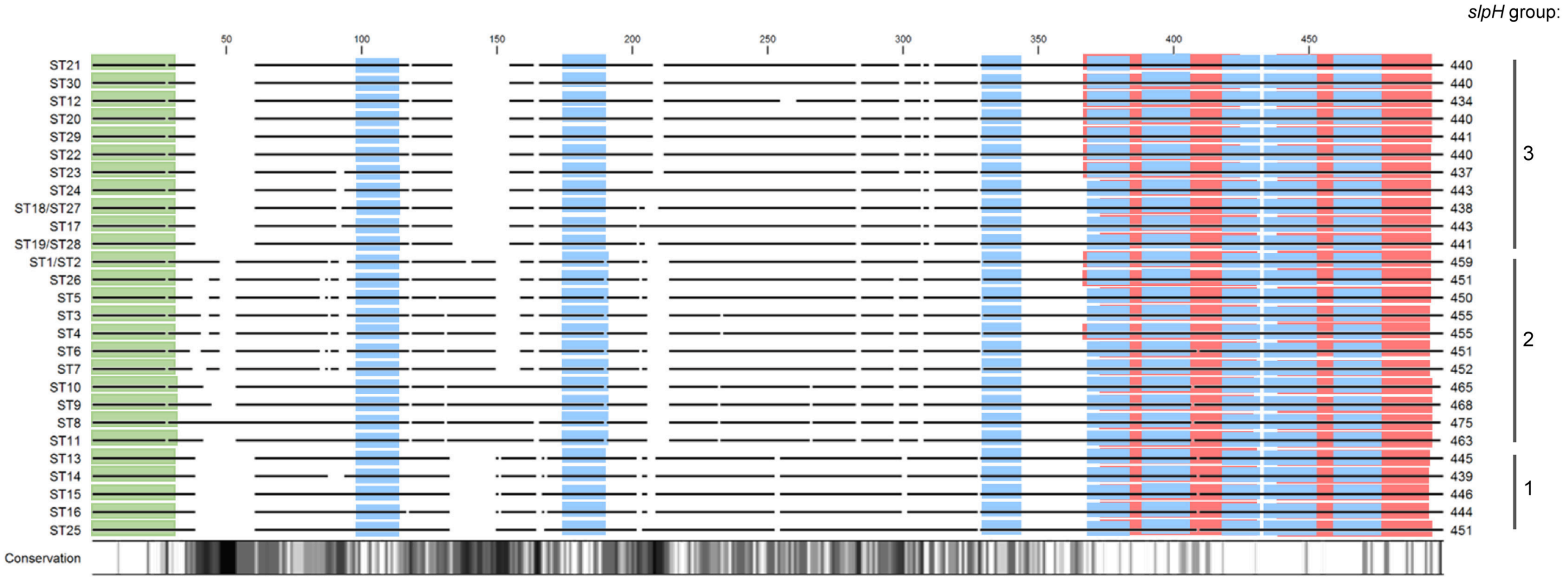
Alignment of the deduced amino acid sequences from the *slpH* genes from all of the different sequence types (STs). The solid lines represent aligned amino acid sequences. Gaps between the lines represent gaps in the alignment. The length of the amino acid sequence is given for all of the STs. The degree of amino acid conservation among the different sequences is shown with a gradient ranging from white to black. White regions indicate 100% conservation, whereas black regions indicate 0% conservation. The proteins were compared to each other *in silico* using InterProScan. The red horizontal bars indicate the tandem SLAP domain (InterPro accession number IPR024968). The blue and green bars represent conserved motifs (PRINTS) of the *Lactobacillus* surface layer protein family (InterPro accession number PR01729) and the signal peptide domains, respectively.

When the deduced amino acid sequences were compared, the primary sequences of ST1 and ST2, ST18 and ST27, and ST19 and ST28, respectively, were identical. All other STs could still be discriminated based on amino acid sequence and especially a high level of variation was observed between the three *slpH* groups (Figure 2). When the primary sequences were analyzed for domains, InterProScan analysis assigned all STs but ST8, ST9, ST10, and ST11, which belong to the slpH2 group, to the *Lactobacillus* surface layer protein family (InterPro accession number PIRSF037863) using the PIRSF family classification system (not shown). Furthermore, InterProScan identified in all STs the eight motif fingerprints of *Lactobacillus* surface layer protein family (InterPro accession number PR01729) based on the PRINTS database for protein fingerprints (blue bars, Figure 2). Additionally, the analysis predicted the presence of a tandem SLAP domain (InterPro accession number IPR024968) in all STs (red bars, Figure 2). Finally, a signal peptide with a length of 30 amino acids was predicted for all the STs (green bars, Figure 2).

**Figure 3:**
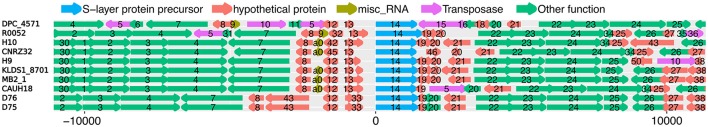
Comparison of the *slpH* gene (14) and surrounding genes of 10 *L. helveticus* strains. The complete genome sequences were retrieved from GenBank. Orthologous gene clusters were determined using Ortho-MCL. Genes belonging to the same orthologous gene cluster are depicted as arrows with the same number. The different colors represent different gene functions.

When we compared the surrounding context of the *slpH* locus in 10 strains possessing different STs and *slpH* groups, we found that up- and downstream various OGCs were co-localized with the *slpH* locus between the strains (Figure 3). Nine of the genes surrounding *slpH* were present in all of the analyzed strains (Figure 3).

### Evidence for the absence of *L. gallinarum* in dairy products

Since the primer pair LHslpF/R can also amplify a region from the gDNA of *L. gallinarum* strains, we established a PCR to assay dairy products for the presence of this species. A primer pair targeting the *pheS* gene of *L. gallinarum* showed species-specificity, as only a 180-bp amplicon was amplified from the gDNA of *L. gallinarum*, but not from closely related LAB (Table 4). The assay's limit of detection was determined by analyzing gDNA extracted from a dilution series of *L. gallinarum* DSM 10532. The lower limit of detection was determined to be at 2.45 × 10^2^ colony forming units per mL broth. The method was generally applied to all samples used for semiconductor sequencing to confirm the absence of *L. gallinarum*.

### Experimental validation

A mixture of 10 *L. helveticus* gDNAs, each of which represented a different ST (Table 3), was prepared. The amplicons obtained after *slpH* amplification were sequenced on the Ion Torrent PGM sequencer. After extraction of the reads containing the *slpH* group specific identifying subsequences, the proportion of reads assigned to the 10 known STs ranged from 5.85 (±0.12)% to 15.73 (±1.00)% (Table 3). Each *slpH* group also comprised reads that were not assigned to the known STs and indicate the presence of new STs. The abundance of those reads per new ST did not exceed 1.7 (±0.3)% of all reads per *slpH* group. Based on this observation, we decided that at least 3% of all reads within a *slpH* group must be assigned to an ST to be considered a true positive result.

### Comparison culture-dependent vs. culture-independent typing

**Figure 4:**
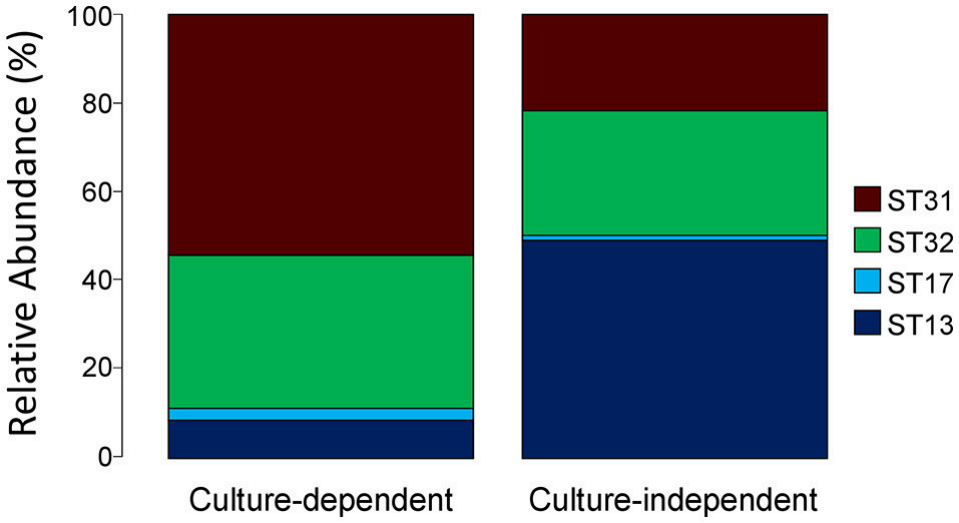
Comparison of the culture-dependent and culture-independent approaches. A natural whey culture (NWC) sample from La Praz, Switzerland was analyzed by plating. The *slpH* gene sequences were determined from 81 *L. helveticus* colonies by Sanger sequencing. The same NWC sample was examined using amplicon-based semiconductor sequencing without prior bacterial isolation. The stacked barplots show the relative abundance of each sequence type (ST) found in the sample for both methods.

We also assessed the applicability of the *slpH* locus for typing, by comparing the *slpH* loci obtained after strain isolation using the culture-dependent approach with those obtained with the culture-independent approach. To effect this, the NWC from La Praz (sample p in Table 2) was plated on MRS agar plates containing lactose as a carbohydrate source. Of the 96 colonies picked, 81 were identified as *L. helveticus* using a species-specific qPCR. The *slpH* locus was amplified and sequenced from each *L. helveticus* isolate. In addition to two previously known STs, we identified two new STs, 31 and 32. The four STs were distributed as follows: 54.3% of the colonies were assigned to ST13 (*slpH* group 1), 8.6% were assigned to ST31 (*slpH* group 2), and 34.6 and 2.5% were assigned to ST17 and ST32, respectively (both *slpH* group 3; Figure 4).

This result was then compared to the sequence analysis obtained from the culture-independent approach. We identified the same STs as found by the culture-dependent approach. All assigned reads were distributed as follows: 21.66% were assigned to ST13, 49.04% were assigned to ST31, and 28.23 and 1.07% were assigned to ST17 and ST32, respectively (Figure 4).

### Distribution of *L. helveticus* strains in NWCs

**Figure 5:**
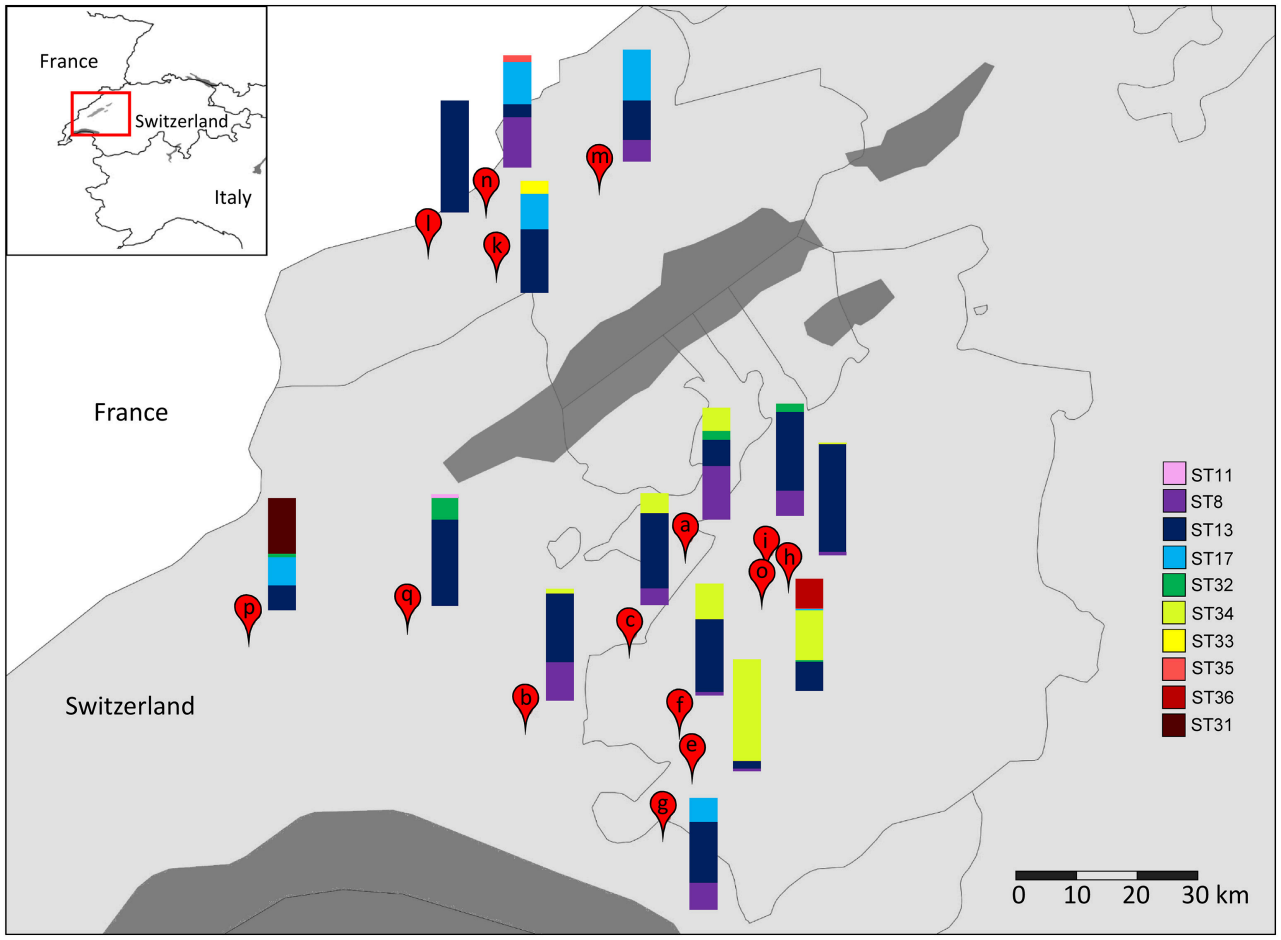
Geographical distribution of *L. helveticus* strains. The diversity of *slpH* loci present in NWC samples collected from cheese factories in (a) Villarzel, (b) Corcelles-le-Jorat, (c) Brenles, (e) Semsales, (f) Le Crêt, (g) Châtel-St. Denis, (h) Autigny, (i) Chénens, (k) Les Monts de Travers, (l) La Brévine, (m) La Sagne, (n) Le Cerneux-Péquignot, (o) Orsonnens, (p) La Praz, and (q) Penthéréaz were analyzed by amplicon-based semiconductor sequencing. Borders between provinces and countries are indicated by black lines. Dark gray areas indicate lakes. The relative abundance (%) of each sequence type (ST) is represented by the height of the relative color in the stacked barplot for each sample.

The diversity of *L. helveticus* strains present in 15 NWCs, collected at variously located Gruyère cheesemaking factories (Table 2), was analyzed using our established culture-independent method. The quantification of *L. helveticus* using qPCR revealed an average population density of 4.42 (±3.79) × 10^7^ cells per mL of NWCs according to *pheS* copy numbers. The presence of *L. gallinarum* was not detected by the specific PCR assay. When we analyzed the *slpH* loci using the culture-independent approach, we detected a total of 10 STs in the 15 NWC samples (Figure 5). The presence of STs ranged from one to five per NWC. Therefore, for example, the NWC from La Brévine contained only one strain, whereas the specimen from Orsonnens contained five STs. The majority (10 NWCs) contained three different STs of *L. helveticus*. Remarkably, ST13 was present in all the NWC samples (Figure 5, blue bar).

### Development of *L. helveticus* strains in raclette cheeses

**Figure 6:**
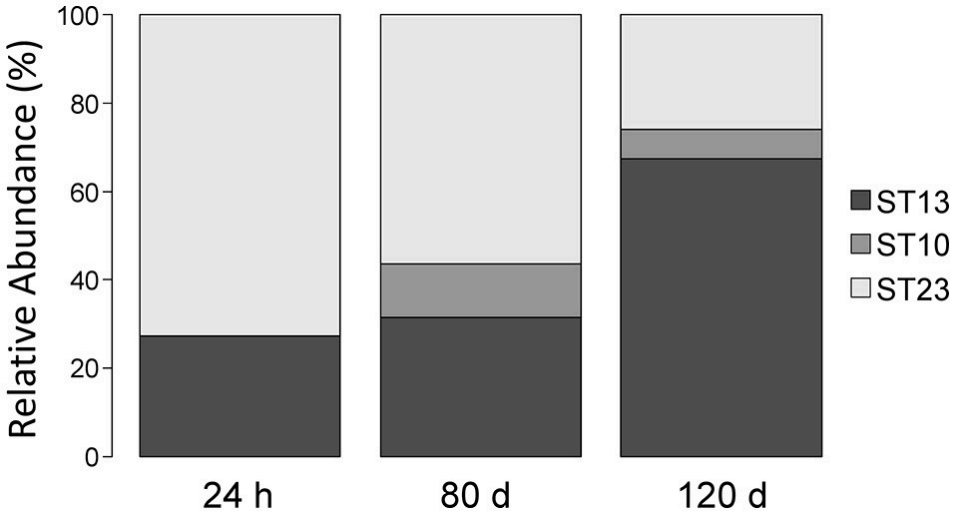
Relative abundance of *L. helveticus* strains during cheese ripening. Raclette cheese was made with three different sequence types (STs) of *L. helveticus;* samples were taken after 24 h, 80 d, and 120 d of ripening. The relative abundance of each ST was determined for each sample using the herein developed culture-independent approach for strain typing.

We also monitored the development of *L. helveticus* strains during cheese ripening by analyzing samples collected from a cheese that had been manufactured with three *L. helveticus* STs, namely ST10 (FAM13019), ST 23 (FAM23236), and ST13 (FAM23237). QPCR estimated the population density of *L. helveticus* to be 10^8^ copies g^−1^ cheese after 24 h of ripening and 10^6^ copies g^−1^ cheese after 80–120 days (Table 2). By using the typing method, we detected only two of the three added *L. helveticus* STs in the cheese ripened for 24 h (Figure 6). After 80 and 120 days, all three of the STs used were detected. The relative abundance of ST13 increased from 27.36% after 24 h of ripening to 31.42% after 80 days and 67.47% after 120 days. For ST23, the relative abundance decreased from 72.64% after 24 h of ripening to 56.34% after 80 days and 25.92% after 120 days. ST10 was not detected in the cheese after 24 h of ripening and had a relative abundance of 12.24% after 80 days, which decreased to 6.6% after 120 days (Figure 6).

## Discussion

The use of high-throughput sequencing techniques enables DNA-based surveys on the biodiversity of food ecosystems without the need to cultivate bacteria. To our knowledge, we present the first study to analyze *L. helveticus* strain diversity occurring in dairy products using next-generation sequencing. By analyzing the genomic sequences of 79 *L. helveticus* strains—58 own-sequenced genomes and 21 genomes taken from the GenBank database—, we found that the *slpH* gene exhibits a high amount of nucleotide polymorphisms and could be a suitable target for amplicon based high-throughput sequencing.

The nucleotide sequence heterogeneity of this locus in *L. helveticus* has already been described by other researchers (Ventura et al., 2000; Gatti et al., 2005; Waśko et al., 2014). Since Waśko et al. (2014) reported they could not detect the *slpH* gene in five *L. helveticus* strains, the question arises if this gene is ubiquitous in *L. helveticus*. Unfortunately, the authors neither explained how the species of these strains were determined nor did they show experimental evidence for the lack of the *slpH* gene in their paper. In contrast, we found that the *slpH* gene is part of the *L. helveticus* core genome using the 79 genomic sequences.

With regard to the nucleotide sequence heterogeneity present in the surface layer protein encoding genes, Gatti et al. (2005) found that the gene sequences clustered in two groups. The gene showed either similarity to the *slpH1* gene encoding a surface layer protein (NCBI accession number X9119) or to the *prtY* gene encoding a putative cell surface proteinase (NCBI accession number AB026985). The *slpH1* and *prtY* group correspond to the *slpH* groups 3 and 2 identified in this study (Figure 1). Additionally, we found a third group named *slpH* group 1 in this report. BLAST searches with the nucleotide sequences of this group resulted in putative surface layer proteins (data not shown). Despite these considerable sequence variations with ambiguous BLAST search results, we think that all sequences are alleles of the same locus. First, bioinformatics analyses showed that the deduced amino acid sequences of the three *slpH* groups clustered in the same OGC. Second, analysis of the genomic context of the *slpH* locus revealed conserved gene neighborhood. Finally, the search for domains revealed the presence of conserved amino acid motifs of the *Lactobacillus* surface layer protein family in all of the analyzed sequences. It is noteworthy that the protein sequence analysis of lactobacilli surface layer proteins was proposed as a method for strain typing (Podleśny et al., 2011). The authors demonstrated that the primary sequence variability present in the surface layer proteins could be determined with LC-MS/MS. Consequently, the method was suitable for lactobacilli strain identification within the *L. acidophilus* group.

The polymorphisms present in the *slpH* gene allowed us to differentiate 30 of the 79 strains used in this study. Conserved nucleotide sequences enabled us the design of a primer pair, which amplified a part of all known *slpH* loci. These primers were not totally species-specific, since an amplicon was also observed in *L. gallinarum*, the closest relative of *L. helveticus*. Although *L. gallinarum* has been detected in cheese by Van Hoorde et al. (2008), this species is usually associated with animals and has been isolated mainly from chicken guts (Hagen et al., 2003; Hammes and Hertel, 2006). Therefore, the occurrence of *L. gallinarum* in dairy products is probably of minor importance, since it is related to contamination. Further studies, such as metagenomics analyses of cheese or the use of *L. gallinarum* and *L. helveticus* in cheese experiments, will clarify whether dairy products are actually a habitat for *L. gallinarum*. Despite these shortcomings, we developed a species-specific PCR assay to analyze dairy products for the presence of *L. gallinarum*.

Due to the next-generation sequencing technology used is this study, we had to breakdown the amplicons that ranged between 1,104 and 1,230 bp. Consequently, only reads possessing the identifying subsequences could be used to detect STs (Supplemental Figure S1). Using this information we still differentiate of 24 STs of the 79 study strains and detected two new STs in the NWC of La Praz. New developments in next-generation sequencing technologies with larger read lengths will enable the use of the complete amplicon sequence for typing.

The applicability of the method was tested with a defined *L. helveticus* mixture, NWCs and cheese. In case of the *L. helveticus* mixture, all expected strains were identified. With regard to the NWC, no differences in the strain composition were found when a culture-dependent analysis was performed. A remarkable result was that in most cases, the *L. helveticus* population in NWCs was composed of more than one *L. helveticus* strain, often with representatives from all three *slpH* groups. Also in the cheese experiment all *L. helveticus* strains used for production were found. Again it was remarkable that the three strains coexisted after 120 days of ripening (Figure 4).

We assume that these strains are different in the phenotype. The coexistence in cheese could result from nutrient or physicochemical gradients present in this habitat. Possible reasons for strain diversity in whey could be caused by variations in whey composition over time or by the fact that some cheesemakers of Gruyère cheese use a mixture of whey cultures that are cultivated under different conditions. Another explanation for the stable coexistence of several strains is the reciprocal loss of metabolic genes resulting in inter-dependencies between strains, as discussed by Ellegaard and Engel (2016). Furthermore, bacteriophages might play an important role in the maintenance of bacterial strain diversity (Ellegaard and Engel, 2016). Currently, the drivers for intra-species diversity are not fully understood and need further investigations. NWCs or cheeses can serve as model microbial ecosystems to study the evolution of microbial diversity, as has been suggested by Wolfe and Dutton (2015).

## Conclusions

Various cheese types (e.g., Italian-hard cheeses, Gruyère PDO cheese) are produced using NWCs. These starter cultures are undefined and *L. helveticus* has been shown to be one of the predominant species. The method presented herein was suitable for determining the biodiversity of *L. helveticus* present in NWCs, without the need to isolate single strains. It was also used to study the development of *L. helveticus* strains during cheese ripening. Cheesemakers of Swiss cheese varieties often assume that *L. helveticus* is the cause of unwanted openings, such as splits and cracks, in cheese. Our established culture-independent approach can be used to verify this hypothesis by studying the development of *L. helveticus* strains from the beginning of the cheesemaking process until the end of cheese ripening. Since *L. helveticus* is also widely used as starter culture in cheese making, the method can be used to monitor strains during cheese ripening and reveal relationships of certain strains with desired cheese properties. Therefore, we consider that the method is useful for studying the development and diversification of *L. helveticus* strain communities in cheese and to better understand its influence on cheese quality.

## Author contributions

AM, SI, LM, and EE: Conceived and designed the study; AM: Performed experiments; DW and RB: Performed bioinformatics analyses; AM, SI, and DW: wrote the manuscript; LM and EE: contributed to the final manuscript.

### Conflict of interest statement

The authors declare that the research was conducted in the absence of any commercial or financial relationships that could be construed as a potential conflict of interest.
