# Supplementary material for: Amplicon Sequencing of the slpH Locus Permits Culture-Independent Strain Typing of Lactobacillus helveticus in Dairy Products
Source: Front Microbiol. 2017 Jul 20;8:1380. doi: 10.3389/fmicb.2017.01380 (PMC5517455; doi:10.3389/fmicb.2017.01380)
Supplement: Supplementary file 1 [file Table1.DOCX]

**Table S1**

*Lactobacillus helveticus* strains from the Agroscope culture collection used for genome sequencing

| **Strain** | **Origin** | **sequence type** | **slpH group** |
| --- | --- | --- | --- |
| FAM1172 | NA | ST13 | 1 |
| FAM1213 | NA | ST22 | 3 |
| FAM1450 | NA | ST20 | 3 |
| FAM1476 | NA | ST1 | 2 |
| FAM1479 | NA | ST1 | 2 |
| FAM8101 | Tilsit cheese | ST13 | 1 |
| FAM8102 | Tilsit cheese | ST13 | 1 |
| FAM8103 | Tilsit cheese | ST13 | 1 |
| FAM8104 | Tilsit cheese | ST13 | 1 |
| FAM8105 | Tilsit cheese | ST13 | 1 |
| FAM8106 | Tilsit cheese | ST13 | 1 |
| FAM8107 | Tilsit cheese | ST13 | 1 |
| FAM8108 | Appenzell cheese | ST13 | 1 |
| FAM8109 | Appenzell cheese | ST13 | 1 |
| FAM8110 | Appenzell cheese | ST13 | 1 |
| FAM8111 | Appenzell cheese | ST13 | 1 |
| FAM11051 | MSS | ST15 | 1 |
| FAM13019 | NWC | ST10 | 2 |
| FAM14274 | NA | ST13 | 1 |
| FAM14275 | NA | ST13 | 1 |
| FAM14276 | NA | ST13 | 1 |
| FAM14278 | NA | ST13 | 1 |
| FAM14279 | NA | ST13 | 1 |
| FAM14280 | NA | ST13 | 1 |
| FAM14498 | NA | ST13 | 1 |
| FAM14499 | NA | ST13 | 1 |
| FAM14500 | NA | ST13 | 1 |
| FAM17275 | NA | ST9 | 2 |
| FAM19188 | Tilsit cheese | ST13 | 1 |
| FAM19189 | Tilsit cheese | ST13 | 1 |
| FAM19190 | Gruyère cheese | ST13 | 1 |
| FAM19191 | Gruyère cheese | ST13 | 1 |
| FAM20575 | Gruyère cheese | ST8 | 2 |
| FAM21340 | MSS | ST14 | 1 |
| FAM21341 | MSS | ST14 | 1 |
| FAM21346 | MSS | ST14 | 1 |
| FAM21348 | MSS | ST14 | 1 |
| FAM21456 | MSS | ST15 | 1 |
| FAM21462 | MSS | ST15 | 1 |
| FAM21463 | MSS | ST15 | 1 |
| FAM21493 | MSS | ST14 | 1 |
| FAM21790 | MSS | ST17 | 3 |
| FAM22077 | NWC | ST3 | 2 |
| FAM22078 | NWC | ST3 | 2 |
| FAM22079 | NWC | ST3 | 2 |
| FAM22080 | MSS | ST1 | 2 |
| FAM22156 | NWC | ST11 | 2 |
| FAM22157 | NWC | ST13 | 1 |
| FAM22192 | NWC | ST21 | 3 |
| FAM22243 | NWC | ST13 | 1 |
| FAM22287 | NWC | ST21 | 3 |
| FAM22292 | NWC | ST11 | 2 |
| FAM22330 | MSS | ST17 | 3 |
| FAM23235 | NA | ST23 | 3 |
| FAM23236 | NA | ST23 | 3 |
| FAM23237 | Tilsit cheese | ST13 | 1 |
| FAM23285 | Tilsit cheese | ST13 | 1 |
| LH-32 | DVS | ST27 | 3 |

Abbreviations are: NWC, natural whey culture; MSS, mixed strain culture; DVS, direct vat set culture (Chr. Hansen Holding A/S, Denmark) NA, information about isolation source not available
