# Supplementary material for: Amplicon Sequencing of the slpH Locus Permits Culture-Independent Strain Typing of Lactobacillus helveticus in Dairy Products
Source: Front Microbiol. 2017 Jul 20;8:1380. doi: 10.3389/fmicb.2017.01380 (PMC5517455; doi:10.3389/fmicb.2017.01380)
Supplement: Supplementary file 2 [file Image1.PDF]

```
                LHslpF          Start codon
CNRZ32 CAAGGAGGAAAGACCACATGAAGAAAAATTTAAGAATTGTTAGCGCTGCTGCTGCTGCTTTATTAGCTGTTGCACCTATTGCTGCAACTGCTATGC---CT
ST21   CAAGGAGGAAAGACCACATGAAGAAAAATTTAAGAATTGTTAGCGTTGCTGCTGCTGCTTTATTAGCTGTTGCTCCTGTTGCTGCAACTGCTATGC---CT
ST30   CAAGGAGGAAAGACCACATGAAGAAAAATTTAAGAATTGTTAGCGCTGCTGCTGCTGCTTTATTAGCTGTTGCACCTATTGCTGCAACTGCTATGC---CT
ST12   CAAGGAGGAAAGACCACATGAAGAAAAATTTAAGAATTGTTAGCGCTGCTGCTGCTGCTTTATTAGCTGTTGCACCTATTGCTGCAACTGCTATGC---CT
ST20   CAAGGAGGAAAGACCACATGAAGAAAAATTTAAGAATTGTTAGCGTTGCTGCTGCTGCTTTATTAGCTGTTGCTCCTGTTGCTGCAACTGCTATGC---CT
ST29   CAAGGAGGAAAGACCACATGAAGAAAAATTTAAGAATTGTTAGCGTTGCTGCTGCTGCTTTATTAGCTGTTGCTCCTGTTGCTGCAACTGCTATGC---CT
ST22   CAAGGAGGAAAGACCACATGAAGAAAAATTTAAGAATTGTTAGCGTTGCTGCTGCTGCTTTATTAGCTGTTGCTCCTGTTGCTGCAACTGCTATGC---CT
ST23   CAAGGAGGAAAGACCACATGAAGAAAAATTTAAGAATTGTTAGCGCTGCTGCTGCTGCTTTATTAGCTGTTGCTCCTGTTGCTGCAACTGCTATGC---CT
ST24   CAAGGAGGAAAGACCACATGAAGAAAAATTTAAGAATTGTTAGCGCTGCTGCTGCTGCTTTATTAGCTGTTGCTCCTGTTGCTGCAACTGCTATGC---CT
ST27   CAAGGAGGAAAGACCACATGAAGAAAAATTTAAGAATTGTTAGCGCTGCTGCTGCTGCTTTATTAGCAGTTGCACCTATTGCTGCAACTGCTATGC---CT
ST17   CAAGGAGGAAAGACCACATGAAGAAAAATTTAAGAATTGTTAGCGCTGCTGCTGCTGCTTTATTAGCTGTTGCTCCTGTTGCTGCAACTGCTATGC---CT
ST19   CAAGGAGGAAAGACCACATGAAGAAAAATTTAAGAATTGTTAGCGTTGCTGCTGCTGCTTTATTAGCTGTTGCTCCTGTCGCTGCTTCTGCTGTAAGCACT
ST28   CAAGGAGGAAAGACCACATGAAGAAAAATTTAAGAATTGTTAGCGTTGCTGCTGCTGCTTTATTAGCTGTTGCTCCTGTCGCTGCTTCTGCTGTAAGCACT
ST1    CAAGGAGGAAAGACCACATGAAGAAAAATTTAAGAATTGTTAGCGCTGCTGCTGCTGCTTTATTAGCTGTTGCACCTATTGCTGCAACTGCTATGC---CA
ST2    CAAGGAGGAAAGACCACATGAAGAAAAATTTAAGAATTGTTAGCGCTGCTGCTGCTGCTTTATTAGCTGTTGCACCTATTGCTGCAACTGCTATGC---CA
ST26   CAAGGAGGAAAGACCACATGAAGAAAAATTTAAGAATTGTTAGCGCTGCTGCTGCTGCTTTATTAGCTGTTGCTCCTGTTGCTGCAACTGCTATGC---CA
ST5    CAAGGAGGAAAGACCACATGAAGAAAAATTTAAGAATTGTTAGCGCTGCTGCTGCTGCTTTATTAGCTGTTGCTCCTGTTGCTGCAACTGCTATGC---CA
ST3    CAAGGAGGAAAGACCACATGAAGAAAAATTTAAGAATTGTTAGCGCTGCTGCTGCTGCTTTATTAGCTGTTGCACCTATTGCTGCAACTGCTATGC---CA
ST4    CAAGGAGGAAAGACCACATGAAGAAAAATTTAAGAATTGTTAGCGCTGCTGCTGCTGCTTTATTAGCAGTTGCACCTATTGCTGCAACTGCTATGC---CA
ST6    CAAGGAGGAAAGACCACATGAAGAAAAATTTAAGAATTGTTAGCGCTGCTGCTGCTGCTTTATTAGCTGTTGCTCCTGTTGCTGCAACTGCTATGC---CA
ST7    CAAGGAGGAAAGACCACATGAAGAAAAATTTAAGAATTGTTAGCGTTGCTGCTGCTGCTTTATTAGCTGTTGCTCCTGTTGCTGCAACTGCTATGC---CA
ST10   CAAGGAGGAAAGACCACATGAAGAAAAATTTAAGAATTGTTAGCGCTGCTGCTGCTGCTTTATTAGCTGTTGCACCTATTGCTGCAACTGCTATGC---CA
ST9    CAAGGAGGAAAGACCACATGAAGAAAAATTTAAGAATTGTTAGCGCTGCTGCTGCTGCTTTATTAGCTGTTGCACCTATTGCTGCAACTGCTATGC---CA
ST8    CAAGGAGGAAAGACCACATGAAGAAAAATTTAAGAATTGTTAGCGCTGCTGCTGCTGCTTTATTAGCTGTTGCACCTATTGCTGCAACTGCTATGC---CA
ST11   CAAGGAGGAAAGACCACATGAAGAAAAATTTAAGAATTGTTAGCGCTGCTGCTGCTGCTTTATTAGCTGTTGCACCTATTGCTGCAACTGCTATGC---CA
ST13   CAAGGAGGAAAGACCACATGAAGAAAAATTTAAGAATTGTTAGCGCTGCTGCTGCTGCTTTATTAGCTGTTGCACCTATTGCTGCAACTGCTGTATCTACT
ST14   CAAGGAGGAAAGACCACATGAAGAAAAATTTAAGAATTGTTAGCGCTGCTGCTGCTGCTTTATTAGCTGTTGCACCTATTGCTGCAACTGCTGTATCTACT
ST15   CAAGGAGGAAAGACCACATGAAGAAAAATTTAAGAATTGTTAGCGCTGCTGCTGCTGCTTTATTAGCTGTTGCACCTATTGCTGCAACTGCTGTATCTACT
ST16   CAAGGAGGAAAGACCACATGAAGAAAAATTTAAGAATTGTTAGCGCTGCTGCTGCTGCTTTATTAGCTGTTGCACCTATTGCTGCAACTGCTGTATCTACT
ST25   CAAGGAGGAAAGACCACATGAAGAAAAATTTAAGAATCGTTAGCGTTGCTGCTGCTGCTTTATTAGCAGTTGCTCCTGTTGCTGCAACTGCTGTATCTACT

CNRZ32 GTTAATGCTGCAACTACTATTAACGCTG---------------------------------------------------ATTCAGCTATCAA----------
ST21   GTTAATGCTGCAACTACTATTAACGCTG---------------------------------------------------ATTCAGCTATCAA----------
ST30   GTTAATGCTGCAACTACTATTAACGCTG---------------------------------------------------ATTCAGCTATCAA----------
ST12   GTTAATGCTGCAACTACTATTAACGCTG---------------------------------------------------ATTCAGCTATCAA----------
ST20   GTTAATGCTGCAACTACTATTAACGCTG---------------------------------------------------ATTCAGCTATCAA----------
ST29   GTTAATGCTGCAACTACTATTAACGCTG---------------------------------------------------ATTCAGCTATCAA----------
ST22   GTTAATGCTGCAACTACTATTAACGCTG---------------------------------------------------ATTCAGCTATCAA----------
ST23   GTTAATGCTGCAACTACTATTAACGCTG---------------------------------------------------ATTCAGCTATCAA----------
ST24   GTTAATGCTGCAACTACTATTAACGCTG---------------------------------------------------ATTCAGCTATCAA----------
ST27   GTTAATGCTGCAACTACTATTAACGCTG---------------------------------------------------ATTCAGCTATCAA----------
ST17   GTTAATGCTGCAACTACTATTAACGCTG---------------------------------------------------ATTCAGCTATCAA----------
ST19   GTTAATGCTGCAACTACTATTAACGCTG---------------------------------------------------GTTCAGCTGTCAA----------
ST28   GTTAATGCTGCAACTACTATTAACGCTG---------------------------------------------------GTTCAGCTGTCAA----------
ST1    GTTAACGCTGCTACTACTGCTACTACTGCTACTACTGC------------------TACTACTTCAACTACTACTAATAAGCCAACTGTTGACTTAAGTGGT
ST2    GTTAACGCTGCTACTACTGCTACTACTGCTACTACTGC------------------TACTACTTCAACTACTACTAATAAGCCAACCGTTGACTTAAGTGGT
ST26   GTTAACGCTGCTACTACTGTTACTACT------------------------------------TCAACTACTACTAACAAGCCAACTGTTGACTTAAGTGGT
ST5    GTTAACGCTGCTACTACTGTTACTACT------------------------------------TCAACTACTACTAACAAGCCAACTGTTGACTTAAGTGGT
ST3    GTTAACGCTGCTACTACTGCTACTACTGCTACTACT---------------------------TCAACTACTACTAATAAGCCAACTGTTGACTTAAGTGGT
ST4    GTTAACGCTGCTACTACTGCTACTACTGCTACTACT---------------------------TCAACTACTACTAATAAGCCAACTGTTGACTTAAGTGGT
ST6    GTTAACGCTGCTACTACTGCTACTGCTACTGCT------------------------------TCAACTACTACTAACAAGCCAACTGTTGACTTAAGTGGT
ST7    GTTAACGCTGATACTACTGCTACTGCTACTG---------------------------------CAACTACTACTAACAAGCCAACTGTTGACTTAAGTGGT
ST10   GTTAACGCTGCAACTACTGCTACTACTGCAACTACTGC---------TA---------------------------ACAAGCCAACTGTTGACTTAAGTGGT
ST9    GTTAACGCTGCAACTACTGCTACTACTGCAACTACTGCAACTACTGCTA---------------------------ACAAGCCAACTGTTGACTTAAGTGGT
ST8    GTTAACGCTGCAACTACTGCTACTACTGCTACTACTGCAACTACTGCTACTACTGCTACTACTGCAACTACTGCTAACAAGCCAACTGTTGACTTAAGTGGT
ST11   GTTAACGCTGCAACTACTGCTACTACTGCAACTACTGC------------------------------------TAACAAGCCAACTGTTGACTTAAGTGGT
ST13   GTTTCTGCTGCTACTACAGTTAATATCGAC-------------------------------------------------GGTAACACTTCAACACCAGTTGC
ST14   GTTTCTGCTGCTACTACAGTTAATATCGAC-------------------------------------------------GGTAACACTTCAACACCAGTTGC
ST15   GTTTCTGCTGCTACTACAGTTAATATCGAC-------------------------------------------------GGTAACACTTCAACACCAGTTGC
ST16   GTTTCTGCTGCTACTACAGTTAATATCGAC-------------------------------------------------GGTAACACTTCAACACCAGTTGC
ST25   GTTTCTGCTGCTACTACAGTTAATATCGAC-------------------------------------------------GGTAACACTTCAACACCAGTTGC

CNRZ32 -----TACTAATACTAATGCTAAGTACGATGTTGACGTAACTCCAAG---CATATCAGCTATTGCTAAAGTTATTGGTACTGGTATTA------CTATCCCA
ST21   -----TGCTAATACTAATGCTAAGTACGATGTTGACGTAACTCCAAG---CATATCAGCTATTGCTGCAGTTGCTAAGAGCGATACTATGCCAGCTATCCCA
ST30   -----TGCTAATACTAATGCTAAGTACGATGTTGACGTAACTCCAAG---CATATCAGCTATTGCTGCAGTTGCTAAGAGCGATACTATGCCAGCTATCCCA
ST12   -----TGCTAATACTAATGCTAAGTACGATGTTGACGTAACTCCAAG---CATATCAGCTATTGCTGCAGTTGCTAAGAGCGATACTATGCCAGCTATCCCA
ST20   -----TGCTAATACTAATGCTAAGTACGATGTTGACGTAACTCCAAG---CATATCAGCTATTGCTGCAGTTGCTAAGAGCGATACTATGCCAGCTATCCCA
ST29   -----TGCTAATACTAATGCTAAGTACGATGTTGACGTAACTCCAAG---CATATCAGCTATTGCTGCAGTTGCTAAGAGCGATACTATGCCAGCTATCCCA
ST22   -----TGCTAATACTAATGCTAAGTACGATGTTGACGTAACTCCAAG---CATATCAGCTATTGCTGCAGTTGCTAAGAGCGATACTAAGCCAGCTATCCCA
ST23   -----TGCTAATACTAATGCTAAGTACGATGTTGACGTAACTCCAAG---CATATCAGCTATTGCTAAAGTTACTGGTAGCGCTACTA---------TCCCA
ST24   -----TGCTAATACTAATGCTAAGTACGATGTTGACGTAACTCCAAG---CATATCAGCTATTGCTAAAGTTACTGGTAGCGCTACTA---------TCCCA
ST27   -----TACTAATACTAATGCTAAGTACGATGTTGACGTAACTCCAAG---CATATCAGCTATTGCTAAAGTTATTGGTACTGGTATTA------CTATCCCA
ST17   -----TACTAATACTAATGCTAAGTACGATGTTGACGTAACTCCAAG---CATATCAGCTATTGCTAAAGTTATTGGTACTGGTATTA------CTATCCCA
ST19   -----TACTAATACTAATGCTAAGTACGATGTTGACGTAACTCCAAG---CGTATCAGCTATTGCTAAAGTTGCTGGCAGCGATACTATGCCAACTATCCCA
ST28   -----TACTAATACTAATGCTAAGTACGATGTTGACGTAACTCCAAG---CGTATCAGCTATTGCTAAAGTTGCTGGTAGCGATACTATGCCAACTATCCCA
ST1    GCAGGTTCTGTTAGTGAATCTAAGGATACTGTTAATGTAACTCCATCATTTACTTTGACTAGTGCTATTCCTGCT---AAGGGTGTT------------CCT
ST2    GCAGGTTCTGTTAGTGAATCTAAGGATACTGTTAATGTAACTCCATCATTTACTTTGACTAGTGCTATTCCTGCT---AAGGGTGTT------------CCT
ST26   GCAGGTTCTGTTAGTGAATCTAAGGATATTGTTAATGTAACTCCATCATTTACTTTGACTAGTGCT------GCT---AAGGGTATT------------CCT
ST5    GCAGGTTCTGTTAGTGAATCTAAGGATACTGTTAATGTAACTCCATCATTTACTTTGATTAGTGCT------GCT---AAGGGTATT------------CCT
ST3    GCAGGTTCTGTTAGTGAATCTAAGGATACTGTTAATGTAACTCCATCATTTACTTTGACTAGTGCTATTCCTGCT---AAGGGTGTT------------CCT
ST4    GCAGGTTCTGTTAGTGAATCTAAGGATACTGTTAATGTAACTCCATCATTTACTTTGACTAGTGCTATTCCTGCT---AAGGGTGTT------------CCT
ST6    GCAGGTTATGTTAGTGAATCTAAGGATATTGTTAATGTAACTCCATCATTTACTTTGACTAGTGCT------GCT---AAGGGTATT------------CCT
ST7    GCAGGTTCTGTTAGTGAATCTAAGGATACTGTTAAGGTAACTCCATCATTTACTTTGAATAGTGCTA------CT---AAGAGTACT------------CCT
ST10   GCAGGTTCTGTTAGTGAATCTAAGGATACTGTTAATGTAACTCCATCATTTACTTTGACTAGTGCTATTGCTACTGATAAGGGTGTAGTT---ACTACGCCT
ST9    GCAGGTTCTGTTAGTGAATCTAAGGATACTGTTAATGTAACTCCATCATTTACTTTGACTAGTGCTATTGCTACTGATAAGGGTGTAGTT---ACTACGCCT
ST8    GCAGGTTCTGTTAGTGAATCTAAGGATACTGTTAATGTAACTCCATCATTTACTTTGACTAGTGCTATTGCTACTGATAAGGGTGTAGTT---ACTACGCCT
ST11   GCAGGTTCTGTTAGTGAATCTAAGGATACTGTTAATGTAACTCCATCATTTACTTTGACTAGTGCTATTGCTACTGATAAGGGTGTAGTT---ACTACGCCT
ST13   ACAA-----AATACAGATGTAAACCTTGCTACTAACTTTACTGCA---------------ATCGCTTATGTTGCAGGTCAAAACGGTACTCAAGGTACAAAC
ST14   ACAA-----AATACAGATGTAAACCTTGCTACTAACTTTACTGCA---------------ATCGCTTATGTTGCAGGT------------------ACAAAC
ST15   ACAA-----AATACAGATGTAAACCTTGCTACTAACTTTACTGCA---------------ATCGCTTATGTTGCAGGTCAAAACGGTACTCAAGGTACAAAC
ST16   ACAA-----AATACAGATGTAAACCTTGCTACTAACTTTACTGCA---------------ATCGCTTATGTTGCAGGTCAAAACGGTACTCAAGGTACAAAC
ST25   ACAA-----AATACAGATGTAAACCTTGCTACTAACTTTACTGCA---------------ATCGCTTATGTTGCAGGTCAAAATGGTCAAGAAGGTACAAAA
```

```
CNRZ32 GGTAGCCTTACTGGTAGTATTTCAGCAAGTTACAATGGTAAGTCATACACTGCTAACTTACCAAAGGATTCAGAAAATGCTACTATTACCAATAG-------
ST21 GGTAGCCTTACTGGTAGTATTTCAGCAAGTTACAATGGTAAGTCATACACTGCTAACTTACCAAAGGATTCAGGAAATGCTACTATTACCGATAG-------
ST30 GGTAGCCTTACTGGTAGTATTTCAGCAAGTTACAATGGTAAGTCATACACTGCTAACTTACCAAAGGATTCAGGAAATGCTACTATTACCGATAG-------
ST12 GGTAGCCTTACTGGTAGTATTTCAGCAAGTTACAATGGTAAGTCATACACTGCTAACTTACCAAAGGATTCAGGAAATGCTACTATTACCGATAG-------
ST20 GGTAGCCTTACTGGTAGTATTTCAGCAAGTTACAATGGTAAGTCATACACTGCTAACTTACCAAAGGATTCAGGAAATGCTACTATTACCGATAG-------
ST29 GGTAGCCTTACTGGTAGTATTTCAGCAAGTTACAATGGTAAGTCATACACTGCTAACTTACCAAAGGATTCAGGAAATGCTACTATTACCGATAG-------
ST22 GGTAGCCTTACTGGTAGTATTTCAGCAAGTTACAATGGTAAGTCATACACTGCTAACTTACCAAATGATTCAGGAAATGCTACTATTACCGATAG-------
ST23 GGTAGCCTTACTGGTAGTATTTCAGCAAGTTACAATGGTAAGTCATACACTGCTAACTTACCAAAGGATTCAGGAAATGCTACTATTACCGATAG-------
ST24 GGTAGCCTTACTGGTAGTATTTCAGCAAGTTACAATGGTAAGTCATACACTGCTAACTTACCAAAGGATTCAGGAAATGCTACTATTGCCGATAA-------
ST27 GGTAGCCTTACTGGTAGTATTTCAGCAAGTTACAATGGTAAGTCATACACTGCTAACTTACCAAAGGATTCAGAAAATGCTACTATTACCAATAG-------
ST17 GGTAGCCTTACTGGTAGTATTTCAGCAAGTTACAATGGTAAGTCATACACTGCTAACTTACCAAAGGATTCAGAAAATGCTACTATTACCAATAG-------
ST19 GGTAGCCTTACTGGTAGTATTTCAGCAAGTTACAATGGTAAGTCATACACTGCTAACTTAGCAAAGGATACAAAAAATGCTACTATTAGCGATGG-------
ST28 GGTAGCCTTACTGGTAGTATTTCAGCAAGTTACAATGGTAAGTCATACACTGCTAACCTAGCAAAGGATACAAAAAATGCTACTATTAGCGATGG-------
ST1 GCAACTCTTCAAGGTAGTATTGAAGCTTCTCTTAACGGTACTTCAGTGACTGCTGATGTAGCTGACGTTGCTAAAGATGTTACCTTGACAGATAG-------
ST2 GCAACTCTTCAAGGTAGTATTGAAGCTTCTCTTAACGGTACTTCAGTGACTGCTGATGTAGCTGACGTTGCTAAAGATGTTACCTTGACAGATAG-------
ST26 GCAACTCTTCAAGGTAGTATTGAAGCTTCTCTTAACGGTACTTCAGTGACTGCTGATGTAGCTGACGTTGCTAAAGATGTTACCTTGACAGATGA-------
ST5 GCAACTCTTCAAGGTAGTATTGAAGCTTCTCTTAACGGTACTTCAGTGACTGCTGATGTAGCTGACGTTGCTAAAAATGTTACCTTGACAGATG--------
ST3 GCAACTCTTCAAGGTAGTATTGAAGCTTCTCTTAACGGTACTTCAGTGACTGCTGATGTAGCTGACGTTGCTAAAGGTGTTACCTTGACAGATGG-------
ST4 GCAACTCTTCAAGGTAGTATTGAAGCTTCTCTTAACGGTACTTCAGTGACTGCTGATGTAGCTGACGTTGCTAAAGGTGTTACCTTGACAGATGG-------
ST6 GCAACTCTTCAAGGTAGTATTGAAGCTTCTCTTAACGGTACTTCAGTGACTGCTGATGTAGCTGACGTTGCTAAAGGTGTTACCTTGACAGATGG-------
ST7 GCAACTCTTAAAGGTAGTATTGAAGCTTCTCTTAACGGTACTTCAGTGACTGCTGATATAGCTGACGTTGCTAAAGATGTTACCTTGAAAGATGA-------
ST10 GCAACTCTTAAGGGTAGTATTGAAGCTTCTCTTAACGGTACTTCAGTGACTGCTGATGTAGCTGACGTTGCTAAAGATGTTACCTTGAAAAATGG-------
ST9 GCAACTCTTAAGGGTAGTATTGAAGCTTCTCTTAACGGTACTTCAGTGACTGCTGATGTAGCTGACGTTGCTAAAGATGTTACCTTGAAAAATGG-------
ST8 GCAACTCTTAAGGGTAGTATTGAAGCTTCTCTTAACGGTACTTCAGTGACTGCTGATGTAGCTGACGTTGCTAAAGATGTTACCTTGAAAAATGG-------
ST11 GCAACTCTTAAGGGTAGTATTGAAGCTTCTCTTAACGGTACTTCAGTGACTGCTGATGTAGCTGACGTTGCTAAAGATGTTACCTTGAAAAATGG-------
ST13 GGTGTTGTTTCAGGTTCTGTAACTGCAACTTACAATGGTCAAAGTTACACTGGTAACTTAACTGATGGTAATACTAAGGATACTACTATCTACAGAGCTTCA
ST14 GGTGTTGTTTCAGGTTCTGTAACTGCAACTTACAATGGTCAAAGTTACACTGGTAACTTAACTGATGGTAATACTAAGGATACTACTATCTACAGAGCTTCA
ST15 GGTGTTGTTTCAGGTTCTGTAACTGCAACTTACAATGGTCAAAGTTACACTGGTAACTTAACTGATGGTAATACTAAGGATACTACTATCTACAGAGCTTCA
ST16 GGTGTTGTTTCAGGTTCTGTAACTGCAACTTACAATGGTCAAAGTTACACTGGTAACTTAACTG---GTAATATTAAGGATACTACTATCTACAGAGCTTCA
ST25 GGTGTTGTTTCAGGTTCTGTAACTGCAACTTACAATGGTCAAAGTTACACTGGCAACTTAACTCATGGTAATGCTAGTGATACTGCTATCTACAGGGTTTCA

CNRZ32 --TAATAATGAGACTGTT-----------------------AAGCCAGCTGATT---------------------------------------TAGAATAT
ST21 --TAATAATAATACTGTT-----------------------AAGCCAGCTGAAT---------------------------------------TAGAAGCT
ST30 --TAATAATAATACTGTT-----------------------AAGCCAGCTGAAT---------------------------------------TAGAAGCT
ST12 --TAATAATAATACTGTT-----------------------AAGCCAGCTGAAT---------------------------------------TAGAAGCT
ST20 --TAATAATAATACTGTT-----------------------AAGCCAGCTGAAT---------------------------------------TAGAAGCT
ST29 --TAATAATAATACTGTT-----------------------AAGCCAGCTAAAT---------------------------------------TAGAAGCT
ST22 --TAATAATAATACTGTT-----------------------AAGCCAGCTGAAT---------------------------------------TAGAAGCT
ST23 --TAATAATAATACTGTT-----------------------AAGCCAGCTGAAT---------------------------------------TAGAAGCT
ST24 --GCATGGTAACCCTGTT-----------------------AAGCCAGCTGATT---------------------------------------TAGAAGCT
ST27 --TAATAATGAGACTGTT-----------------------AAGCCAGCTGATT---------------------------------------TAGAATAT
ST17 --TAATAATGAGACTGTT-----------------------AAGCCAGCTGATT---------------------------------------TAGAATAT
ST19 --TAGTAATAATAATGTT-----------------------GATCCAGCTGATT---------------------------------------TAGAATAT
ST28 --TAGTAATAATAATGTT-----------------------GATCCAGCTGATT---------------------------------------TAGAATAT
ST1 --TAAGGGTGTTGCTGTTTACAGTTACGATAAAGAC------AAGCTTACTAATAAGCTTAGTG---------------------------ATGTAAAAGCA
ST2 --TAAGGGTGTTGCTGTTTACAGTTACGATAAAGAC------AAGCTTACTAATAAGCTTAGTGAT-------------------------GTAAAAGCA
ST26 --TGGTGGCAAGACTGTTTACAGTTACAATGACCGCGAAAATAAGGTTGATAATAACCTTAGTGCT-------------------------GTAGAAGCA
ST5 ----GTAACAAGACTGTTTACAGTTACAATGAACGCGAAAATAAGGTTGATAATAACCTTAGTGAT-------------------------GTACAAGCA
ST3 --TAACAAGA---CTGTTTACAGTTACAATGAACACGAAAATAAGGTTGATAATAACCTTAGTGCT-------------------------GTAGAAGCA
ST4 --TAACAAGC---CTGTTTACAGTTACAATGAACACGAAAATAAGGTTGATAATAACCTTAGTGCT-------------------------GTAGAAGCA
ST6 --TAACAAGA---CTGTTTACAGTTACAATGAACGCGAAAATAAGGTTGATAATAACCTTAGTGCT-------------------------GTAGAAGCA
ST7 --TGGTGGCAAGACTGTTTACAGTTACAATGAAGGCGAAAAGAAGGTTGATAATAACCTTAGTGCT-------------------------GTAGAAGCA
ST10 --TGACACGG---TTGTTTACAGTTACGATGCAAAAAACAATAAGCCTATTAATAACTTGGGTGCTTATGACACATCTGCTAAGAAAGCATATGTAGAAGCA
ST9 --TGACACGG---TTGTTTACAGTTACGATGCAAAAAACAATAAGCCTATTAATAACTTGGGTGCTTATGACACATCTGCTAAGAAAGCATATGTAGAAGCA
ST8 --TGACACGG---TTGTTTACAGTTACGATGCAAAAAACAATAAGCCTATTAATAACTTGGGTGCTTATGACACATCTGCTAAGAAAGCATATGTAGAAGCA
ST11 --TGACACGG---TTGTTTACAGTTACGATGCAAAAAACAATAAGCCTATTAATAACTTGGGTGCTTATGACACATCTGCTAAGAAAGCATATGTAGAAGCA
ST13 GATAAAAAACCT---GTT----------------------GAGGTTAGTTCAAA-----------------------------------------AGACTTT
ST14 GATAAAAAACCT---GTT----------------------GAGGTTAGTTCAAA-----------------------------------------AGACTTT
ST15 GATAAAAAAACT---GTT----------------------GAGGTTAGTTCAAA-----------------------------------------AGACTTT
ST16 GATAAAAAACAT---GTT----------------------GAGGTTAGTTCAAA-----------------------------------------AGACTTT
ST25 GATAAAAGACATCATGTT----------------------GATGTTAGTTCACC-----------------------------------------AGCATTT

CNRZ32 GGCAAGC------CTTACAAAGTAACTGTTCCTGATGTTTCATTTAACTTTGGTTCAGAAAATGCAGGTAAGGAAATTACTATTGGTTCAGCTAACCAAAAT
ST21 GACAAGG------CTTACACAGTAACTGTTCCTGATGTTTCATTTAACTTTGGTTCAGAAAATGCAGGTAAGGAAATTACTATTGGTTCAGCTAACCCAAAT
ST30 GACAAGG------CTTACACAGTAACTGTTCCTGATGTTTCATTTAACTTTGGTTCAGAAAATGCAGGTAAGGAAATTACTATTGGTTCAGCTAACCCAAAT
ST12 GACAAGG------CTTACACAGTAACTGTTCCTGATGTTTCATTTAACTTTGGTTCAGAAAATGCAGGTAAGGAAATTACTATTGGTTCAGCTAACCCAAAT
ST20 GACAAGG------CTTACACAGTAACTGTTCCTGATGTTTCATTTAACTTTGGTTCAGAAAATGCAGGTAAGGAAATTACTATTGGTTCAGCTAACCCAAAT
ST29 GACAAGG------CTTACACAGTAACTGTTCCTGATGTTTCATTTAACTTTGGTTCAGAAAATGCAGGTAAGGTAATTACTATTGGTTCAGCTAACCCAAAT
ST22 GACAAGG------CTTACACAGTAACTGTTCCTGATGTTTCATTTAACTTTGGTTCAGAAAATGCAGGTAAGGAAATTACTATTGGTTCAGCTAACCCAAAT
ST23 GACAAGG------CTTACACAGTAACTGTTCCTGATGTTTCATTTAACTTTGGTTCAGAAAATGCAGGTAAGGAAATTACTATTGGTTCAGCTAACCCAAAT
ST24 GACAAGG------CTTACACAGTAACTGTTCCTGATGTTTCATTTAACTTTGGTTCAGAAAATGCAGGTAAGGAAATTACTATTGGTTCAGCTAACCAAAAT
ST27 GGCAAGC------CTTACAAAGTAACTGTTCCTGATGTTTCATTTAACTTTGGTTCAGAAAATGCAGGTAAGGAAATTACTATTGGTTCAGCTAACCAAAAT
ST17 GGCAAGC------CTTACAAAGTAACTGTTCCTGATGTTTCATTTAACTTTGGTTCAGAAAATGCAGGTAAGGAAATTACTATTGGTTCAGCTAACCAAAAT
ST19 GGCAAGC------CTTACACAGTAACTGTTCCTGATGTTTCATTTAACTTTGGTTCAGAAAATGCAGGTAAGGAAATTACTATTGGTTCAGCTAACCAAAAT
ST28 GGCAAGC------CTTACACAGTAACTGTTCCTGATGTTTCATTTAACTTTGGTTCAGAAAATGCAGGTAAGGAAATTACTATTGGTTCAGCTAACCAAAAT
ST1 GGTGATGA------CTACACTATGACTCTTAGTGGTGTAGGCTTCAGCTTTGGTAAAGCTAATGCAGGCAAGACCTTAACTTTTAAGTTGCCTAA---GAAT
ST2 GGTGATGA------CTACACTATGACTCTTAGTGGTGTAGGCTTCAGCTTTGGTAAAGCTAATGCAGGCAAGACCTTAACTTTTAAGTTGCCTAA---GAAT
ST26 GGTAAGAA------ATACACTATGACTCTTAGTGGTGTAGGCTTCAGCTTTGGTAAAGCTAATGCAGGCAAGACCTTAACTTTTAAGTTGCCTAA---GAAT
ST5 AGTAAAGA------ATACACTATGACTCTTAGTGGTGTAGGCTTCAGCTTTGGTAAAGCTAATGCAGGCAAGACCTTAACTTTTAAGTTGCCTGA---GAAT
ST3 GGTAAAGA------ATACACTATGACTCTTAGTGGTGTAGGCTTCAGCTTTGGTAAAGCTAATGCAAAAAAGACCTTAACTTTTAAGTTGCCTAA---AGGT
ST4 GGTAAAGA------ATACACTATGACTCTTAGTGGTGTAGGCTTCAGCTTTGGTAAAGCTAATGCAAACAAGACCTTAACTTTTAAGTTGCATAA---TGGT
ST6 AGTAAAGA------ATACACTATGACTCTTAGTGGTGTAGGTTTCAGCTTTGGTAAAGCTAATGCAGGCAAGACCTTAACTTTTAAGTTGCCTAA---GAAT
ST7 GGTAAGGA------ATACACTATGACTCTTAGTGGTGTAGGCTTCAGCTTTGGTAAAGCTAATGCAGGCAAGACCTTAACTTTTAAGTTGCCTAA---GAAT
ST10 AATAAAAATAAATCATACACTATGACTCTTAGTGGTGTAGGCTTCAGCTTTGGTAAAGCTAATGCAGGCAAGACCTTAACTTTCAAGTTGCCTAC---AGAT
ST9 AATAAAAATAAATCATACACTATGACTCTTAGTGGTGTAGGCTTCAGCTTTGGTAAAGCTAATGCAGGCAAGACCTTAACTTTCAAGTTGCCTAC---AGAT
ST8 AATAAA------TCATACACTATGACTCTTAGTGGTGTAGGCTTCAGCTTTGGTAAAGCTAATGCAGGCAAGACCTTAACTTTCAAGTTGCCTAC---AGAT
ST11 AATAAA------TCATACACTATGACTCTTAGTGGTGTAGGCTTCAGCTTTGGTAAAGCTAATGCAGGCAAGACCTTAACTTTCAAGTTGCCTAC---AGAT
ST13 GCTGCAGGTCAATACTAC---GCTGTAATTAAAGACGTTTCATTCAACTTTGGTTCACAAAACGCAGATAAGAAGCTCACTGTTAGTCT------TAAGGGT
ST14 GCTGCAGGTCAATACTAC---GCTGTAATTAAAGACGTTTCATTCAACTTTGGTTCACAAAACGCAGATAAGAAGCTCACTGTTAGTCT------TAAGGGT
ST15 GCTGCAGGTCAATACTACTACGCTGTAATTAAAGACGTTTCATTCAACTTTGGTTCACAAAACGCAGATAAGAAGCTCACTGTTAGTCT------TAAGGGT
ST16 GCTGCAGGTCAATACTAC---GCTGTAATTAAAGATGTTTCATTCAACTTTGGTTCACAAAACGCAGATAAGAAGCTCACTGTTAGTCT------TAAGGGT
ST25 AGTGCAGGTCAATACTGC---GCTAAAATTAGAAACGTTTCATTCAACTTTGGTTCACAAAACGCAGGTAAGAAGCTCACTGTTAGTCT------TTGGGGT
```

NGS typing region, Group 3 identifying subsequences are underlined

NGS typing region, Group 3

```
CNRZ32 GTAACCTTTACTAACAA---TGGCCAA---------------ACTGGTTCAACTGTAAAGGTTAAGTTAGACCAAAATGGTGTTGCTACTCTTTCAAGTGTA
  ST21 GTAACCTTTACTGAAAAGACCGGTGA-------CCAACC-----TGCTTCAACTGTAAAAGTTACGTTAGACCAAGATGGTGTTGCTAAGCTTTCAAGTGTA
  ST30 GTAACCTTTACTGAAAAGACCGGTGA-------CCAACC-----TGCTTCAACTGTAAAAGTTACGTTAGACCAAGATGGTGTTGCTAAGCTTTCAAGTGTA
  ST12 GTAACCTTTACTGAAAAGACCGGTGA-------CCAACC-----TGCTTCAACTGTAAAAGTTACGTTAGACCAAGATGGTGTTGCTAAGCTTTCAAGTGTA
  ST20 GTAACCTTTACTAAAAAGACCGGTGA-------CCAACC-----TGCTTCAACTGTAAAAGTTACGTTAGACCAAGATGGTGTTGCTAAGCTTTCAAGTGTA
  ST29 GTAACCTTTACTAAAAAGACCGGTGA-------CCAACC-----TGCTTCAACTGTAAAAGTTACGTTAGACCAAGATGGTGTTGCTAAGCTTTCAAGTGTA
  ST22 GTAACCTTTACTGAAAAGACCGGTGA-------CCAACC-----TGCTTCAACTGTAAAAGTTACGTTAAACCAAGATGGTGTTGCTAAGCTTTCAAGTGTA
  ST23 GTAACCTTTACTGAAAAGAAAGGTGA-------CCAACC-----TGCTTCAACTGTAAAAGTTACGTTAGACCAAGATGGTGTTGCTAAGCTTTCAAGTGTA
  ST24 GTAACCTTTACTACTAAAGATAGCCAAAGTGGTTCAACTGTAAGTGGTTCAACTGTAAAGGTTACGTTAGACCAAGATGGTGTTGCTAAGCTTTCAAGTGTA
  ST27 GTAACCTTTACTAACAA---TGGCCAA---------------ACTGGTTCAACTGTAAAGGTTAAGTTAGACCAAAATGGTGTTGCTACTCTTTCAAGTGTA
  ST17 GTAACCTTTACTACTAA---AGATAAAGATGGCAAACCTGTAACTGGTCCAACTGCACATGTTAAGTTAGACCAAAATGGTGTTGCTACTCTTTCAAGTGTA
  ST19 GTAACCTTTACTAACAA---TAGCAAA---------------ACTGGTTCAACTGTAAAGGTTACGTTAGACCAAAATGGTGTTGCTACTCTTTCAAGTGTA
  ST28 GTAACCTTTACTAACAA---TAGCAAA---------------ACTGGTTCAACTGTAAAGGTTACGTTAGACCAAAATGGTGTTGCTACTCTTTCAAGTGTA
   ST1 GTTAAAGTTAACGATACTAGCAATGA---------------------------TGTTAAAGTAAGTTTGGACCAATACGGTAATGCAACTAATTTGAAGTTT
   ST2 GTTAAAGTTAACGATACTAGC---AA------------------------TGATGTTAAAGTAAGTTTGGACCAATACGGTAATGCAACTAATTTGAAGTTT
  ST26 GTTAAAGTTAACGATACTAGC---AA------------------------TGATGTTAAAGTAAGTTTGGACCAATACGGTAATGCAACTAATTTGAAGTTT
   ST5 GTTAAAGTTAACGATACTAGC---AA------------------------TGATGTTAAAGTAAGTTTGGACCAATACGGTAATGCAACTAATTTGAAGTTT
   ST3 GTTACAGTTGAAGGTGCTAACTACAA------------------------GGATGGTAAAGTAACTTTGGACCAATACGGTAATGTAACTGATTTGAAGTTT
   ST4 GCTACAGTTGAAGGTGCTAACTACAA------------------------GGATGGTAAAGTAACTTTGGACCAATACGGTAATGTAACTGATTTGAAGTTT
   ST6 GTTAAAGTTAACGATACTAGC---AA------------------------TGAAGTTAAAGTAAGTTTGGACCAATACGGTAATGCAACTAATTTGAAGTTT
   ST7 GTTAAAGTTAACGATACTAGC---AA------------------------TGATGTTAAAGTAAGTTTGGACCAATACGGTAATGCAACTAATTTGAAGTTT
  ST10 GTTCAAGTTTCTGGTGATAACTACAA------------------------GGATGGTAAAGTAACTTTGGACCAATACGGTAATGTAACTGGTTTGAAGTTT
   ST9 GTTCAAGTTTCTGGTGATAACTACAA------------------------GGATGGTAAAGTAACTTTGGACCAATACGGTAATGTAACTGGTTTGAAGTTT
   ST8 GTTCAAGTTTCTGGTGATAACTACAA------------------------GGATGGTAAAGTAACTTTGGACCAATACGGTAATGTAACTGGTTTGAAGTTT
  ST11 GTTCAAGTTTCTGGTGATAACTACAA------------------------GGATGGTAAAGTAACTTTGGACCAATACGGTAATGTAACTGGTTTGAAGTTT
  ST13 GGTTTATTAACTACTACTGACAAAGATGCT---------AAGCCTGCTGAAAGCGTTACTGTAACTTTGGATAAGAATGGTGTTGCAAACTTTGCAGAAGTT
  ST14 GGTTTATTAACTACTACTGACAAAGATGCT---------AAGCCTGCTGAAAGCGTTACTGTAACTTTGGATAAGAATGGTGTTGCAAACTTTGCAGAAGTT
  ST15 GGTTTATTAACTACTACTGACAAAGATGCT---------AAGCCTGCTGAAAGCGTTACTGTAACTTTGGATAAGAATGGTGTTGCAAACTTTGCAAAAGTT
  ST16 GGTTTATTAACTACTACTGACAAAGATGCT---------AAGCCTGCTGAAAGCGTTACTGTAACTTTGGATAAGAATGGTGTTGCAAACTTTGCAGAAGTT
  ST25 GGTTCATTAACTACTCTTAACAGAAAGACTCATCACAGAAGGACTGGTGAAAGCCTTTCTGTAACTTTGGATAAGGATGGTGTTGCAAACTTTGCAGAAGTT

CNRZ32 CAAATTAAGAATGTTTACGCAGTTAACACTACTGACAACAGAGATGTAAACTTCTACGATGTAACAACTGGTGCTACTGTAAAAACTGGTGCTGTTTCTCTT
  ST21 CAAATTAAGAATGTTTACGCAATTGACACTACTTACAACAGCAATGTAAACTTCTACGATGTAACAACTGGTGCTACTGTAACAACTGGTGCTGTTTCTATT
  ST30 CAAATTAAGAATGTTTACGCAATTGACACTACTTACAACAGCAATGTAAACTTCTACGATGTAACAACTGGTGCTACTGTAACAACTGGTGCTGTTTCTATT
  ST12 CAAATTAAGAATGTTTACGCAATTGACACTACTTACAACAGCAATGTAAACTTCTACGATGTAACAACTGGTGCT------------------GTTTCTATT
  ST20 CAAATTAAGAATGTTTACGCAATTGACACTACTTACAACAGCAATGTAAACTTCTACGATGTAACAACTGGTGCTACTGTAACAACTGGTGCTGTTTCTATT
  ST29 CAAATTAAGAATGTTTACGCAATTGACACTACTTACAACAGCAATGTAAACTTCTACGATGTAACAACTGGTGCTATTGTAACAACTGGTGCTGTTTCTATT
  ST22 CAAATTAAGAATGTTTACGCAATTGACACTACTTACAACAGCAATGTAAACTTCTACGATGTAACAACTGGTGCTACTGTAACAACTGGTGCTGTTTCTATT
  ST23 CAAATTAAGAATGTTTACGCAATTGACACTACTTACAACAGCAATGTAAACTTCTACGATGTAACAACTGGTGCTACTGTAACAACTGGTGCTGTTTCTATT
  ST24 CAAATTAAGGATGTTTACGCAATTGACACTACTTACAACAGCAATGTAAACTTCTACGATGTAACAACTGGTGCTATTGTAACAACTGGTGCTGTTTCTATT
  ST27 CAAATTAAGAATGTTTACGCAGTTAACACTACTGACAACAGAGATGTAAACTTCTACGATGTAACAACTGGTGCTACTGTAAAAACTGGTGCTGTTTCTCTT
  ST17 CAAATTAAGAATGTTTACGCAGTTAACACTACTGACAACAGAGATGTAAACTTCTACGATGTAACAACTGGTGCTACTGTAACAACTGGTGCTGTTTCTCTT
  ST19 CAAATTAAGAATGTTTACGCAGTTAACACTACTGACAACAGAGATGTAAACTTCTACGATGTAACAACTGGTGCTACTGTAAAAACTGGTGCTGTTTCTCTT
  ST28 CAAATTAAGAATGTTTACGCAGTTAACACTACTGACAACAGAGATGTAAACTTCTACGATGTAACAACTGGTGCTACTGTAAAAACTGGTGCTGTTTCTCTT
   ST1 GTTATCTCAAACATTAAGGCATATGACTCAGCAAACACTAACGCTGTAAGCTTTTACGATAAGAAGTCAGGCTTAGTTGCAACTCAAGGTTCATACATGACT
   ST2 GTTATCTCAAACATTAAGGCATATGACTCAGCAAACACTAACGCTGTAAGCTTTTACGATAAGAAGTCAGGCTTAGTTGCAACTCAAGGTTCATACATGACT
  ST26 GTTATCTCAAACATTAAGGCATATGACTCAGCAAACACTAACGCTGTAAGCTTTTACGATGCTAAGTCAGGCTTAGTTGCAACTCAAGGTTCATACATGACT
   ST5 GTTATCTCAAAAATTAAGGCATATGACTCAGCAAACACTAACGCTGTAAGCTTTTACGATGCTAAGTCAGGCTTAGTTGCAACTCAAGGTTCATACATGACT
   ST3 ---ACCTTAAAGGTTAAGGCATATGACTCAGCAAACACTAACGCTGTAAGCTTTTACGATAAGAAGTCAGGCTTAGTTGCAACTCAAGGTTCATACATGACT
   ST4 ---ACCTTAAAGGTTAAGGCATATGACTCAGCAAACACTAACGCTGTAAGCTTTTACGATAAGAAGTCAGGCTTAGTTGCAACTCAAGGTTCATACATGACT
   ST6 GTTATCTCAAACATTAAGGCATATGACTCAGCAAACACTAACGCTGTAAGCTTTTACGATGCTAAGTCAGGCTTAGTTGCAACTCAAGGTTCATACATGACT
   ST7 GTTATCTCAAACATTAAGGCATATGACTCAGCAAACACTAACGCTGTAAGCTTTTACGATGCTAAGTCAGGCTTAGTTGCAACTCAAGGTTCATACATGACT
  ST10 ---ACCTTAAACGTTAAGGCATATAACTCAGAAAATACTAACGCTGTAAGCTTTTACGATGCTAAGTCAGGCTTAGTTGCATCTCAAGGTTCATACATGACT
   ST9 ---ACCTTAAACGTTAAGGCATATAACTCAGAAAATACTAACGCTGTAAGCTTTTACGATGCTAAGTCAGGCTTAGTTGCATCTCAAGGTTCATACATGACT
   ST8 ---ACCTTAAACGTTAAGGCATATAACTCAGAAAATACTAACGCTGTAAGCTTTTACGATGCTAAGTCAGGCTTAGTTGCATCTCAAGGTTCATACATGACT
  ST11 ---ACCTTAAACGTTAAGGCATATAACTCAGAAAATACTAACGCTGTAAGCTTTTACGATGCTAAGTCAGGCTTAGTTGCATCTCAAGGTTCATACATGACT
  ST13 CAAACTCCAAACTTTAAGGCTGTAAACCCATTCAGCACTTCAACTGTTGCTTGGTACCA------AAACAATAACGTTGTAACTTCAGCTAACGTAACTGTT
  ST14 CAAACTCCAAACTTTAAGGCTGTAAACCCATTCAGCACTTCAACTGTTGCTTGGTACCA------AAACAATAACGTTGTAACTTCAGCTAACGTAACTGTT
  ST15 CAAACTCCAAACTTTAAGGCTGTAAACCCATTCAGCACTTCAACTGTTGCTTGGTACCA------AAACAATAACGTTGTAACTTCAGCTAACGTAACTGTT
  ST16 CAAACTCCAAACTTTAAGGCTGTAAACCCATTCAGCACTTCAACTGTTGCTTGGTACCA------AAACAATAACGTTGTAACTTCAGCTAACGTAACTGTT
  ST25 CAAACTCCAAACTTTAAGGCTGTAAACCCATTCAGCACTTCAACTGTTGCTTGGTACCA------AAACAATAACGTTGTAACTTCAGCTAACGTAACTGTT

CNRZ32 GACGCTGACAACCAAGGTCAACTTAACACTGCATCTGTTGTAGCTGCAATTACCTCTAA------GTACTTTGCAGCACAATATGCTAATAAGCAATTGTCT
  ST21 GACGCTGACAACCAAGGTCAACTTAACATTACATCTGTTGTAGCTGCAATTAATTCTAA------GTACTTTGCAGCACAATATGATAAGAAACAATTGACT
  ST30 GACGCTGACAACCAAGGTCAACTTAACATTACATCTGTTGTAGCTGCAATTAATTCTAA------GTACTTTGCAGCACAATATGATAAGAAACAATTGACT
  ST12 GACGCTGACAACCAAGGTCAACTTAACATTACATCTGTTGTAGCTGCAATTAATTCTAA------GTACTTTGCAGCACAATATGATAAGAAACAATTGACT
  ST20 GACGCTGACAACCAAGGTCAACTTAACATTACATCTGTTGTAGCTGCAATTAATTCTAA------GTACTTTGCAGCACAATATGATAAGAAACAATTGACT
  ST29 GACGCTGACAACCAAGGTCAACTTAACATTACATCTGTTGTAGCTGCAATTAATTCTAA------GTACTTTGCAGCACAATATGATAAGAAACAATTGACT
  ST22 GACGCTGACAACCAAGGTCAACTTAACATTACATCTGTTGTAGCTGCAATTAATTCTAA------GTACTTTGCAGCACAATATGATAAGAAACAATTGACT
  ST23 GACGCTGACAACCAAGGTCAACTTAACATTACATCTGTTGTAGCTGCAATTAATTCTAA------GTACTTTGCAGCACAATATGATAAGAAACAATTGACT
  ST24 GACGCTGACAACCAAGGTCAACTTAACACTGCATCTGTTGTAGCTGCAATTAGTTCTAA------GTACTTTGCAGCACAATATGCTGATAAGAATTTGACT
  ST27 GACGCTGACAACCAAGGTCAACTTAACACTGCATCTGTTGTAGCTGCAATTACCTCTAA------GTACTTTGCAGCACAATATGCTAATAAGCAATTGTCT
  ST17 GACGCTGACAACCAAGGTCAACTTAACACTGCATCTGTTGTAGCTGCAATTACCTCTAA------GTACTTTGCAGCACAATATGCTAATAAGCAATTGTCT
  ST19 GACGCTGACAACCAAGGTCAACTTAACACTGCATCTGTTGTAGCTGCAATTACCTCTAA------GTACTTTGCAGCACAATATGCTAAGAAGCAATTGTCT
  ST28 GACGCTGACAACCAAGGTCAACTTAACACTGCATCTGTTGTAGCTGCAATTACCTCTAA------GTACTTTGCAGCACAATATGCTAAGAAGCAATTGTCT
   ST1 CTTGCTGATGAAAATGGTAACCTTAATGTAGATGCTCTTTTGAAGGCATTAAATGATAA------GTATGAAGCAATGCAATTCAAAGACGGTAGTTTCCAC
   ST2 CTTGCTGATGAAAATGGTAACCTTAATGTAGATGCTCTTTTGAAGGCATTAAATGATAA------GTATGAAGCAATGCAATTCAAAGACGGTAGTTTCCAC
  ST26 CTTGCTGATGAAAATGGTAACCTTAATGTAACTACTCTTTTGGATAAATTAAAGGGTAA------GTATGAAGCAATGCAATTCAAAGACAGTAAGTTC---
   ST5 CTTGCTGATGAAAATGGTAACCTTAATGTAAATAAGCTTTTGGATGCATTAAAGGGTAA------GTATGAAGCAATGCAATTCCAAAACAGTAAGTTC---
   ST3 CTTGCTGATGAAAATGGTGACCTTAATGTAAATACTCTTTTGGATAAATTAAAGGGTAA------GTATGAAGCAATGCAATTCAAAGACAGTAAGTTT---
   ST4 CTTGCTGATGAAAATGGTAACCTTAATGTAGATGCTCTTTTGAAGGCATTAAATGATAA------GTATGAAGCAATGCAATTCAAAGACAGTAAGTTC---
   ST6 CTTGCTAATGAAAATGGTGACCTTAATGTAGATACTCTTTTGAAGGCATTAAATGGTAA------GTATGAAGCAATGCAATTCCAAAGCGGTAAGTTC---
   ST7 CTTGCTGATGAAAATGGTAACCTTAATGTAAATACTCTTTTGAAGGCATTAAATGATAA------GTATGAAGCAATGCAATTCCGAAACGGTAATTTT---
  ST10 CTTGCTCA---AAATGGTGACCTTAATGTAAATACTCTTTTGAATGAATTAAAGAGTAA------GTATAAAGCAATGCAATTCCAAAACGGTCAGTTC---
   ST9 CTTGCTCA---AAATGGTGACCTTAATGTAAATACTCTTTTGAATGAATTAAAGAGTAA------GTATAAAGCAATGCAATTCCAAAACGGTCAGTTC---
   ST8 CTTGCTCA---AAATGGTGACCTTAATGTAAATACTCTTTTGAATGAATTAAAGAGTAA------GTATAAAGCAATGCAATTCCAAAACGGTAAGTTC---
  ST11 CTTGCTCA---AAATGGTGACCTTAATGTAAATACTCTTTTGAATGAATTAAAGAGTAA------GTATGAAGCAATGCAATTCCAAAACGGTAAGTTC---
  ST13 AACGCAGGTAACAATAACTTGGTTAATGTTAGCCAAATCGTTGCAGCTTTAAACGGCTACACTGCACACGAATTAACTCGTGGTGACAATGGTCAAGTAGTT
  ST14 AACGCAGGTAACAATAACTTGGTTAATGTTAGCCAAATCGTTGCAGCTTTAAACGGCTACACTGCACACGAATTAACTCCTGGTGACAATGGTCAAGTAGTT
  ST15 AACGCAGGTAACAATAACTTGGTTAATGTTAGCCAAATCGTTGCAGCTTTAAACGGCTACACTGCACACGAATTAACTCGTGGTGACAATGGTAAAGTAGAT
  ST16 AACGCAGGTAACAATAACTTGGTTAATGTTAGCCAAATCGTTGCAGCTTTAAACGGCTACACTGCACACGAATTAACTCGTGGTGACAATGGTCAAGTAGGT
  ST25 AACGCAGGTAACAATAACTTGGTTAATGTTAGCCAAATCGTTGCAGCTTTAAACGGCTACACTCCATACGAATTAGCTCGTGGTGACCATGGTAACGTAACT
```

NGS typing region, Group 2 identifying subsequences are underlined

NGS typing region, Group 1 identifying subsequences are underlined

```
CNRZ32 CAAGACA------ATGTTGTAGTTAACACTGAAACTGC---TGTCAAGGATGCTTTAAAGGCTCAAAAGATTGAAGTAAACTCAGTAGGTTAC---TTCAAG
  ST21 AATG------------TTACATTTGACACTGAAACTGC---TGTCAAGGATGCTTTAAAGGCTCAAAAGATTGAAGTAAGCTCAGTAGGTTAC---TTCAAG
  ST30 AATG------------TTACATTTGACACTGAAACTGC---TGTCAAGGATGCTTTAAAGGCTCAAAAGATTGAAGTAAGCTCAGTAGGTTAC---TTCAAG
  ST12 AATG------------TTACATTTGACACTGAAACTGC---TGTCAAGGATGCTTTAAAGGCTCAAAAGATTGAAGTAAGCTCAGTAGGTTAC---TTCAAG
  ST20 AATG------------TTACATTTGACACTGAAACTGC---TGTCAAGGATGCTTTAAAGGCTCAAAAGATTGAAGTAAGCTCAGTAGGTTAC---TTCAAG
  ST29 AATGAT---------GTTACATTTGACACTGAAACTGC---TGTCAAGGATGCTTTAAAGGCTCAAAAGATTGAAGTAAGCTCAGTAGGTTAC---TTCAAG
  ST22 AATG------------TTACATTTGACACTGAAACTGC---TGTCAAGGATGCTTTAAAGGCTCAAAAGATTGAAGTAAGCTCAGTAGGTTAC---TTCAAG
  ST23 AATG------------TTACATTTGACACTGAAACTGC---TGTCAAGGATGCTTTAAAGGCTCAAAAGATTGAAGTAAGCTCAGTAGGTTAC---TTCAAG
  ST24 AGTGATA------ATGTTACATATAACATTGAAACTGC---TGTCAAGGATGCTTTAAAGGCTCAAAAGATTGAAGTAAGCTCAGTAGGTTAC---TTCAAG
  ST27 CAAGACA------ATGTTGTAGTTAACACTGAAACTGC---TGTCAAGGATGCTTTAAAGGCTCAAAAGATTGAAGTAAACTCAGTAGGTTAC---TTCAAG
  ST17 CAAGACA------ATGTTGTAGTTAACACTGAAACTGC---TGTCAAGGATGCTTTAAAGGCTCAAAAGATTGAAGTAAACTCAGTAGGTTAC---TTCAAG
  ST19 CAAGATA------ATGTTGTAGTTAACACTGAAACTGC---TGTCAAGGATGCTTTAAAGGCTCAAAAGATTGAAGTAAACTCAGTAGGTTAC---TTCAAG
  ST28 CAAGATA------ATGTTGTAGTTAACACTGAAACTGC---TGTCAAGGATGCTTTAAAGGCTCAAAAGATTGAAGTAAACTCAGTAGGTTAC---TTCAAG
   ST1 ACTGACAC------TGTTAAGGTAAATACTACTGCTGACGATGTTAAGGCTGAACTTGAAAAGGCTGGTATCAAGGTGGACGCTGCTGGTAAC---TTTGAA
   ST2 ACTGACA------CTGTTAAGGTAAATACTACTGCTGACGATGTTAAGGCTGAACTTGAAAAGGCTGGTATCAAGGTGGACGCTGCTGGTAAC---TTTGAA
  ST26 ---AAAA------CTGTTAATGTAAATACTACTGCTGACGATGTTAAGGCTGAACTTGAAAAGGCTGGTATCAAGGTAGACGCTGCTGGTAAC---TTTGAA
   ST5 ---AAAA------CTGTTAAGGTAAATACTACTGCTGACGATGTTAAGGCTGAACTTGAAAAGGCTGGTATCAAGGTAGACGCTGCTAATAAC---TTTGAA
   ST3 ---GAAA------CTGTTAATGTAAATACTACTCCTGACGATGTTAAGGCTGAACTTGAAAAGGCTGGTATCAAGGTAGACGCTGCTGGTAAC---TTTGAA
   ST4 ---GAAA------CTGTTAATGTAAATACTACTGCTGACGATGTTAAGGCTGAACTTGAAAAGGCTGGTATCAAGGTGGACGCTGCTGGTAAC---TTTGAA
   ST6 ---GCAG------ATGTTACTGTAACTACTAATGCTGACGATGTTAAGGCTGAACTTGAAAAGGCTGGTATCAAGGTAGACGCTGCTAATAAC---TTTGAA
   ST7 ---GCAA------CTGTTACTCCATCTACTACTGCTGACGATGTTAAGGCTGAACTTGAAAAGGCTGGTATCAAGGTAAGCGATGATGGTAGC---TTTGAA
  ST10 ---GAAA------GTGTTACTGTAACTACTACTGCTGACGATGTTAAGGCTGAACTTGAAAAGGCTGGGATCAAGGTAAACGCTGCTGGTGACGACTTTGAA
   ST9 ---GCAA------GTGGTACTGTAACTACTACTGCTGACGATGTTAAGGCTGAACTTGAAAAGGCTGGGATCAAGGTAAACGCTGCTGGTGACGACTTTGAA
   ST8 ---GAAA------GAGTTACTGTAACTACTACTGCTGACGATGTTAAGGCTGAACTTGAAAAGGCTGGGATCAAGGTAAACGCTGCTGGTGACGACTTTGAA
  ST11 ---GAAA------GTGTTACTGTAACTACTACTGCTGACGATGTTAAGGCTGAACTTGAAAAGGCTGGGATCAAGGTAAACGCTGCTGGTGACGACTTTGAA
  ST13 TCAAAC------CCTGTAACTTCACCTATTACTGCTGCTGCAGTTACTGATCAATTAAAGGCACAAAATATTGCTGTTGATGGTGCTGGTTAC---TTTACT
  ST14 TCAAAG------CATGTAACTTCACCTATTACTGCTGCTGCAGTTACTGATCAATTAAAGGCACAAAATATTGCTGTTGATGGTGCTGGTTAC---TTTACT
  ST15 TCAAAC------CCTGTAACTTCACCTATTACTGCTGCTGCAGTTACTGATCAATTAAAGGCACAAAATATTGCTGTTGATGGTGCTGGTTAC---TTTACT
  ST16 TCAAAC------CCTGTAACTTCACCTATTACTGCTGCTGCAGTTACTGATCAATTAAAGGCACAAAATATTGCTGTTGATGGTGCTGGTTAC---TTTACT
  ST25 TCAAGCAATAGCCATTTAACTTCACCTATTACTGCTGCTGCAGTTACTGATCAATTAAAGGCACAAAATATTGCTGTTGATGGTGCTGATTAC---TTTACT

CNRZ32 GCTCCACATACTTTCACTGTTAATGTTAAGGCAACTTCAAACGTTAACAGTAAGTCAGCTACTTTACCAGTAACTGTAACTGTTCCTAACGTTGCAGAACCA
  ST21 GCTCCACATACTTTCACTGTTAATGTTAAGGCAACTTCAAACAAGAACGGTAAGTCAGCTACTTTACCAGTAACTGTAACTGTTCCTAACGTTGCAGATCCT
  ST30 GCTCCACATACTTTCACTGTTAATGTTAAGGCAACTTCAAACAAGAACGGTAAGTCAGCTACTTTACCAGTAACTGTAACTGTTCCTAACGTTGCAGATCCT
  ST12 GCTCCACATACTTTCACTGTTAATGTTAAGGCAACTTCAAACAAGAACGGTAAGTCAGCTACTTTACCAGTAACTGTAACTGTTCCTAACGTTGCAGATCCT
  ST20 GCTCCACATACTTTCACTGTTAATGTTAAGGCAACTTCAAACAAGAACGGTAAGTCAGCTACTTTACCAGTAACTGTAACTGTTCCTAACGTTGCAGATCCT
  ST29 GCTCCACATACTTTCACTGTTAATGTTAAGGCAACTTCAAACAAGAACGGTAAGTCAGCTACTTTACCAGTAACTGTAACTGTTCCTAACGTTGCAGATCCT
  ST22 GCTCCACATACTTTCACTGTTAATGTTAAGGCAACTTCAAACAAGAACGGTAAGTCAGCTACTTTACCAGTAACTGTAACTGTTCCTAACGTTGCAGATCCT
  ST23 GCTCCACATACTTTCACTGTTAATGTTAAGGCAACTTCAAAAAAGAACGGTAAGTCAGCTACTTTACCAGTAACTGTAACTGTTCCTAACGTTGCAGATCCT
  ST24 GCTCCACATACTTTCACTGTTAATGTTAAGGCAACTTCAAACAAGAACGGTAAGTCAGCTACTTTACCAGTAACTGTAACTGTTCCTAACGTTGCAGATCCT
  ST27 GCTCCACATACTTTCACTGTTAATGTTAAGGCAACTTCAAACGTTAACAGTAAGTCAGCTACTTTACCAGTAACTGTAACTGTTCCTAACGTTGCAGAACCA
  ST17 GCTCCACATACTTTCACTGTTAATGTTAAGGCAACTTCAAACGTTAACAGTAAGTCAGCTACTTTACCAGTAACTGTAACTGTTCCTAACGTTGCAGAACCA
  ST19 GCTCCACATACTTTCACTGTTAATGTTAAGGCAACTTCAAACGTTAACAGTAAGTCAGCTACTTTACCAGTAACTGTAACTGTTCCTAACGTTGCAGAACCA
  ST28 GCTCCACATACTTTCACTGTTAATGTTAAGGCAACTTCAAACGTTAACAGTAAGTCAGCTACTTTACCAGTAACTGTAACTGTTCCTAACGTTGCAGAACCA
   ST1 GCACCAGACACATTCACTGTAACTTTAAATGCTAAGTCAGACGTTAACGGTAAGACTGCTTCATTACCTGTAGTTGTAACTGTTCCAAATGGTAAGTCAACT
   ST2 GCACCAGACACATTCACTGTAACTTTAAATGCTAAGTCAGACGTTAACGGTAAGACTGCTTCATTACCTGTAGTTGTAACTGTTCCAAATGGTAAGTCAACT
  ST26 GCACCAGACACATTCACTGTAACTTTAAATGCTAAGTCAGACGTTAACGGTAAGACTGCTCCATTACCTGTAGTTGTAACTGTTCCAAATGGTAAGTCAACT
   ST5 GCACCAGACACATTCACTGTAACTTTAAATGCTAAGTCAGACGTTAACGGTAAGACTGCTTCATTACCTGTAGTTGTAACTGTTCCAAATGGTAAGTCAACT
   ST3 GCACCAGACACATTCACTGTAACTTTAAATGCTAAGTCAGACGTTAACGGTAAGACTGCTTCATTACCTGTAGTTGTAACTGTTCCAAATGGTAAGTCAACT
   ST4 GCACCAGACACATTCACTGTAACTTTAAATGCTAAGTCAGACGTTAACGGTAAGACTGCTTCATTACCTGTAGTTGTAACTGTTCCAAATGGTAAGTCAACT
   ST6 GCACCAGACACATTCACTGTAACTTTAAATGCTAAGTCAGACGTTAACGGTAAGACTGCTTCATTACCTGTAGTTGTAACTGTTCCAAATGGTAAGTCAACT
   ST7 GCACCAGACACATTCACTGTAACTTTAAATGCTAAGTCAGACGTTAACGGTAAGACTGGTTCATTACCTGTAGTTGTAACTGTTCCAAATGGTAAGTCAACT
  ST10 GCACCAGACACATTCACTGTAACTTTAAATGCTAAGTCAGACGATAACGGTAAGACTGCTTCATTACCTGTAGTTGTAACTGTTCCAAATGGTAAGTCAACT
   ST9 GCACCAGACACATTCACTGTAACTTTAAATGCTAAGTCAGACGATAACGGTAAGACTGCTTCATTACCTGTAGTTGTAACTGTTCCAAATGGTAAGTCAACT
   ST8 GCACCAGACACATTCACTGTAACTTTAAATGCTAAGTCAGACGATAACGGTAAGACTGCTTCATTACCTGTAGTTGTAACTGTTCCAAATGGTAAGTCAACT
  ST11 GCACCAGACACATTCACTGTAACTTTAAATGCTAAGTCAGACGATAACGGTAAGACTGCTTCATTACCTGTAGTTGTAACTGTTCCAAATGGTAAGTCAACT
  ST13 GCACCAACTTCATTAAGCTTGAAATTTACTGCTACTGCAAACAATAGTAATGCTTCTGCTGAATTACCAGTTACTGTTTCAATTCCAAACGGTAAGGTAACT
  ST14 GCACCAACTTCATTAAGCTTGAAATTTACTGCTACTGCAAACAATAGTAATGCTTCTGCTGAATTACCAGTTACTGTTTCAATTCCAAACGGTAAGGTAACT
  ST15 GCACCAACTTCATTAAGCTTGAAATTTACTGCTACTGCAAACAATAGTAATGCTTCTGCTGAATTACCAGTTACTGTTTCAATTCCAAACGGTAAGGTAACT
  ST16 GCACCAACTTCATTAAGCTTGAAATTTACTGCTACTGCAAACAATAGTAATGCTTCTGCTGAATTACCAGTTACTGTTTCAATTCCAAACGGTAAGGTAACT
  ST25 GCACCAACTTCATTAAGCTTGAAATTTACTGCTACTGCAGACGATAGTAATGCTTCTGCTGAATTACCAGTTACTGTTTCAATTCCAAACGGTAAGGTAACT

CNRZ32 ACTGTACCAAGTGTATCAAAGACTGTTATGCACAACGCATACTTCTACGACAAGAACGCTAAGCGTGTTGGTACTGACAAGGTAACTCGTTACGACACTGTA
  ST21 GTTGTTCCAAGTCAAAGCAAGACTATTATGCACAACGCTTACTTCTACGACAAGGACGCTAAGCGTGTTGGTACTGACAAGGTAACTCGTTACAACACTGTA
  ST30 GTTGTTCCAAGTCAAAGCAAGACTATTATGCACAACGCTTACTTCTACGACAAGGACGCTAAGCGTGTTGGTACTGACAAGGTAACTCGTTACAACACTGTA
  ST12 GTTGTTCCAAGTCAAAGCAAGACTATTATGCACAACGCTTACTTCTACGACAAGGACGCTAAGCGTGTTGGTACTGACAAGGTAACTCGTTACAACACTGTA
  ST20 GTTGTTCCAAGTCAAAGCAAGACTATTATGCACAACGCTTACTTCTACGACAAGGACGCTAAGCGTGTTGGTACTGACAAGGTAACTCGTTACAACACTGTA
  ST29 GTTGTTCCAAGTCAAAGCAAGACTATTATGCACAACGCTTACTTCTACGACAAGGACGCTAAGCGTGTTGGTACTGACAAGGTAACTCGTTACAACACTGTA
  ST22 GTTGTTCCAAGTCAAAGCAAGACTATTATGCACAACGCTTACTTCTACGACAAGGACGCTAAGCGTGTTGGTACTGACAAGGTAACTCGTTACAACACTGTA
  ST23 GTTGTTCCAAGTCAAAGCAAGACTATTATGCACAACGCTTACTTCTACGACAAGGACGCTAAGCGTGTTGGTACTGACAAGGTAACTCGTTACAACACTGTA
  ST24 GTTGTTCCAAGTCAAAGCAAGACTATTATGCACAACGCATACTTCTACGACAAGGACGCTAAGCGTGTTGGTACTGACAAGGTAACTCGTTACAACACTGTA
  ST27 ACTGTACCAAGTGTATCAAAGACTGTTATGCACAACGCATACTTCTACGACAAGAACGCTAAGCGTGTTGGTACTGACAAGGTAACTCGTTACGACACTGTA
  ST17 ACTGTACCAAGTGTATCAAAGACTGTTATGCACAACGCATACTTCTACGACAAGAACGCTAAGCGTGTTGGTACTGACAAGGTAACTCGTTACAACACTGTA
  ST19 ACTGTACCAAGTGTATCAAAGACTGTTATGCACAACGCATACTTCTACGACAAGAACGCTAAGCGTGTTGGTACTGACAAGGTAACTCGTTACAACACTGTA
  ST28 ACTGTACCAAGTGTATCAAAGACTGTTATGCACAACGCATACTTCTACGACAAGAACGCTAAGCGTGTTGGTACTGACAAGGTAACTCGTTACAACACTGTA
   ST1 GTTGTTCCAAGTCAAAGCAAGACTATTATGCACAACGCATACTTCTACGACAAGAACGCTAAGCGTGTTGGTACTGACAAGGTAACTCGTTACAACGCTGTA
   ST2 GTTGTTCCAAGTCAAAGCAAGACTATTATGCACAACGCATACTTCTACGACAAGAACGCTAAGCGTGTTGGTACTGACAAGGTAACTCGTTACAACGCTGTA
  ST26 GTTGTTCCAAGTCAAAGCAAGACTATTATGCACAACGCATACTTCTACGACAAGAACGCTAAGCGTGTTGGTACTGACAAGGTAACTCGTTACAACGCTGTA
   ST5 GTTGTTCCAAGTCAAAGCAAGACTATTATGCACAACGCATACTACTACGACAAAGACGCTAAGCGTGTTGGTAATGACAAGGTAACTCGTTACAACGCTGTA
   ST3 GTTGTTCCAAGTCAAAGCAAGACTATTATGCACAACGCATACTTCTACGACAAGAACGCTAAGCGTGTTGGTACTGACAAGGTAACTCGTTACAACACTGTA
   ST4 GTTGTTCCAAGTCAAAGCAAGACTATTATGCACAACGCATACTTCTACGACAAGAACGCTAAGCGTGTTGGTACTGACAAGGTAACTCGTTACAACGCTGTA
   ST6 GTTGTTCCAAGTCAAAGCAAGACTATTATGCACAACGCATACTACTACGACAAGGACGCTAAGCGTGTTGGTACTGACAAGTTGACTCGTTACAACTCAGTA
   ST7 GTTGTACCTAGCGTAGCTAAGACTATTATGCACAACGCATACTACTACGACAAGAACGCTAAGCGTGTTGGTACTGACAAGGTAACTCGTTACAACACTGTA
  ST10 GTTGTACCTAGCGCAGCTAAGACTATTATGCACAACGCATACTTCTACGACAAGAACGCTAAGCGTGTTGGTACTGACAAGTTAACTCGTTACAACACTGTA
   ST9 GTTGTACCTAGCGCAGCTAAGACTATTATGCACAACGCATACTTCTACGACAAGAACGCTAAGCGTGTTGGTACTGACAAGTTAACTCGTTACAACACTGTA
   ST8 GTTGTACCTAGCGCAGCTAAGACTATTATGCACAACGCATACTTCTACGACAAGAACGCTAAGCGTGTTGGTACTGACAAGTTAACTCGTTACAACACTGTA
  ST11 GTTGTACCTAGCGCAGCTAAGACTATTATGCACAACGCATACTTCTACGACAAGAACGCTAAGCGTGTTGGTACTGACAAGTTAACTCGTTACAACACTGTA
  ST13 ACTGTTGAAAGCGTATCAAAGACTATTATGCACAACGCATACTACTACGACAAGAACGCTAAGCGTGTTGGTACTGACAAGTTGACTCGTTACAACTCAGTA
  ST14 ACTGTTGAAAGCGTATCAAAGACTATTATGCACAACGCATACTACTACGACAAGAACGCTAAGCGTGTTGGTACTGACAAGTTGACTCGTTACAACTCAGTA
  ST15 ACTGTTGAAAGCGTATCAAAGACTATTATGCACAACGCATACTACTACGACAAGAACGCTAAGCGTGTTGGTACTGACAAGTTGACTCGTTACAACTCAGTA
  ST16 ACTGTTGAAAGCGTATCAAAGACTATTATGCACAACGCATACTACTACGACAAGAACGCTAAGCGTGTTGGTACTGACAAGTTGACTCGTTACAACTCAGTA
  ST25 ACTGTTGAAAGCGTATCAAAGACTATTATGCACAACGCATACTACTACGACAAGAACGCTAAGCGTGTTGGTACTGACAAGGTAACTCGTTACAACACTGTA
```

LHslpR

```
CNRZ32 ACTGTTGCTATGAACACTACTAAGCTTGCTAACGGTATTTCATACTACGAAGTAATCGAAAACGGCAAGGCAACTGGCAAGTACA 1116
ST21   ACTGTTGCTATGAACACTACTAAGCTTGCTAACGGTATTTCATACTACGAAGTAATCGAAAACGGCAAGGCAACTGGCAAGTACA 1122
ST30   ACTGTTGCTATGAACACTACTAAGCTTGCTAACGGTATTTCATACTATGAAGTAATCGAAAACGGCAAGGCAACTGGCAAGTACA 1122
ST12   ACTGTTGCTATGAACACTACTAAGCTTGCTAACGGTATTTCATACTATGAAGTAATCGAAAACGGCAAGGCAACTGGCAAGTACA 1104
ST20   ACTGTTGCTATGAACACTACTAAGCTTGCTAACGGTATTTCATACTACGAAGTAATCGAAAACGGCAAGGCAACTGGCAAGTACA 1122
ST29   ACTGTTGCTATGAACACTACTAAGCTTGCTAACGGTATTTCATACTACGAAGTAATCGAAAACGGCAAGGCAACTGGCAAGTACA 1125
ST22   ACTGTTGCTATGAACACTACTAAGCTTGCTAACGGTATTTCATACTACGAAGTAATCGAAAACGGCAAGGCAACTGGCAAGTACA 1122
ST23   ACTGTTGCTATGAACACTACTAAGCTTGCTAACGGTATTTCATACTACGAAGTAATCGAAAACGGCAAGGCAACTGGCAAGTACA 1113
ST24   ACTGTTGCTATGAACACTACTAAGCTTGCTAACGGTATTTCATACTACGAAGTAATCGAAAACGGCAAGGCAACTGGCAAGTACA 1131
ST27   ACTGTTGCTATGAACACTACTAAGCTTGCTAACGGTATTTCATACTACGAAGTAATCGAAAACGGCAAGGCAACTGGCAAGTACA 1116
ST17   ACTGTTGCTATGAACACTACTAAGCTTGCTAACGGTATTTCATACTACGAAGTAATCGAAAACGGCAAGGCAACTGGCAAGTACA 1131
ST19   ACTGTTGCTATGAACACAACTAAGCTCGCTAACGGTATTTCATACTACGAAGTAATCGAAAACGGCAAGGCAACTGGCAAGTACA 1125
ST28   ACTGTTGCTATGAACACAACTAAGCTCGCTAACGGTATTTCATACTACGAAGTAATCGAAAACGGCAAGGCAACTGGCAAGTACA 1125
ST1    ACTGTTGCTATGAACACTACTAAGCTTGCTAACGGTATTTCATACTACGAAGTAATCGAAAACGGCAAGGCAACTGGCAAGTACA 1179
ST2    ACTGTTGCTATGAACACTACTAAGCTTGCTAACGGTATTTCATACTACGAAGTAATCGAAAACGGCAAGGCAACTGGCAAGTACA 1179
ST26   ACTGTTGCTATGAACACTACTAAGCTTGCTAACGGTATTTCATACTACGAAGTAATCGAAAACGGCAAGGCAACTGGCAAGTACA 1155
ST5    ACTGTTGCTATGAACACTACTAAGCTTGCTAACGGTATTTCATACTACGAAGTAATCGAAAACGGCAAGGCAACTGGCAAGTACA 1152
ST3    ACTGTTGCTATGAACACTACTAAGCTTGCTAACGGTATTTCATACTACGAAGTAATCGAAAACGGCAAGGCAACTGGCAAGTACA 1167
ST4    ACTGTTGCTATGAACACTACTAAGCTTGCTAACGGTATTTCATACTACGAAGTAATCGAAAACGGCAAGGCAACTGGCAAGTACA 1167
ST6    ACTGTTTCACCTAAGACTACTA---CCATCAACGGCAAGGCTTACTACGAAGTAGTTGAAAACGGCAAGGCAACTGGCAAGTACA 1155
ST7    ACTGTTGCTATGAACACTACTAAGCTTGCTAACGGTGTTTCATACTACGAAGTAATCGAAAACGGCAAGGCAACTGGCAAGTACA 1158
ST10   ACTGTTGCAACTTCACCTACTA---TTATCAACGGTAATGCTTACTACGAAGTAATCGAAAACGGCAAGGCAACTGGCAAGTACA 1200
ST9    ACTGTTGCAACTTCACCTACTA---TTATCAACGGTAATGCTTACTACGAAGTAATCGAAAACGGCAAGGCAACTGGCAAGTACA 1209
ST8    ACTGTTGCAACTTCACCTACTA---TTATCAACGGTAATGCTTACTACGAAGTAATCGAAAACGGCAAGGCAACTGGCAAGTACA 1230
ST11   ACTGTTGCAACTTCACCTACTA---TTATCAACGGTAATGCTTACTACGAAGTAATCGAAAACGGCAAGGCAACTGGCAAGTACA 1194
ST13   ACTGTTTCACCTAAGACTACTA---CCATCAACGGCAAGGCTTACTACGAAGTAGTTGAAAACGGCAAGGCAACTGGCAAGTACA 1137
ST14   ACTGTTTCACCTAAGACTACTA---CCATCAACGGCAAGGCTTACTACGAAGTAGTTGAAAACGGCAAGGCAACTGGCAAGTACA 1119
ST15   ACTGTTTCACCTAAGACTACTA---CCATCAACGGCAAGGCTTACTACGAAGTAGTTGAAAACGGCAAGGCAACTGGCAAGTACA 1140
ST16   ACTGTTTCACCTAAGACTACTA---CCATCAACGGCAAGGCTTACTACGAAGTAGTTGAAAACGGCAAGGCAACTGGCAAGTACA 1134
ST25   ACTGTTGCACCTTCAACCACTA---CTATCAACGGTAAGGCTTACTATGAAGTAGTTGAAAACGGCAAGGCAACTGGCAAGTACA 1155
```

Figure S1. Alignment of the nucleotide sequences with all of the identified slpH sequence types. Sequences are aligned to the reference sequence of strain CNRZ32 (NCBI Genbank Accession Number: NC_021744.1). Primer sequences are underlined. The start codon of the slpH gene is marked in a black box. The typing regions for the NGS pipeline are marked in a green box for slpH group 1, in a blue box for slpH group 2, and in a red box for slpH group 3. The identifying subsequences are underlined in the specific color for each slpH group.
